# Biological chromodynamics: a general method for measuring protein occupancy across the genome by calibrating ChIP-seq

**DOI:** 10.1093/nar/gkv670

**Published:** 2015-06-30

**Authors:** Bin Hu, Naomi Petela, Alexander Kurze, Kok-Lung Chan, Christophe Chapard, Kim Nasmyth

**Affiliations:** 1 Department of Biochemistry, University of Oxford, South Parks Road, Oxford OX1 3QU, UK; 2 Department of Molecular Biology and Biotechnology, The University of Sheffield, Western Bank, Sheffield S10 2TN, UK

## Abstract

Sequencing DNA fragments associated with proteins following in vivo cross-linking with formaldehyde (known as ChIP-seq) has been used extensively to describe the distribution of proteins across genomes. It is not widely appreciated that this method merely estimates a protein's distribution and cannot reveal changes in occupancy between samples. To do this, we tagged with the same epitope orthologous proteins in *Saccharomyces cerevisiae* and *Candida glabrata*, whose sequences have diverged to a degree that most DNA fragments longer than 50 bp are unique to just one species. By mixing defined numbers of *C. glabrata* cells (the calibration genome) with *S. cerevisiae* samples (the experimental genomes) prior to chromatin fragmentation and immunoprecipitation, it is possible to derive a quantitative measure of occupancy (the occupancy ratio – OR) that enables a comparison of occupancies not only within but also between genomes. We demonstrate for the first time that this ‘internal standard’ calibration method satisfies the *sine qua non* for quantifying ChIP-seq profiles, namely linearity over a wide range. Crucially, by employing functional tagged proteins, our calibration process describes a method that distinguishes genuine association within ChIP-seq profiles from background noise. Our method is applicable to any protein, not merely highly conserved ones, and obviates the need for the time consuming, expensive, and technically demanding quantification of ChIP using qPCR, which can only be performed on individual loci. As we demonstrate for the first time in this paper, calibrated ChIP-seq represents a major step towards documenting the quantitative distributions of proteins along chromosomes in different cell states, which we term biological chromodynamics.

## INTRODUCTION

Determining what proteins bind to which parts of the genome as cells grow, pass through the cell cycle, and differentiate is vital for understanding how transcription is regulated as well as how chromosomes are replicated and segregated. A widely used technique to do this involves analysis of DNA sequences immunoprecipitated (IP) with defined proteins following formaldehyde fixation and DNA sonication (ChIP-seq) (1,2). This technique has three major limitations. The first is fundamental and probably insuperable. Formaldehyde cross-links single stranded DNA to proteins much more efficiently than it does double stranded DNA. All data obtained by ChIP-seq are therefore prone to artefacts caused by this often ignored fact. This is especially problematical when the technique is used to map the distribution of chromosomal proteins that do not bind to DNA directly and is the reason why ChIP-seq can never obviate the need to observe proteins within living cells (3). The second problem is that as currently practised ChIP-seq merely reveals an estimate of the distribution of a protein across a genome. In other words, it says nothing about the actual occupancy. Given the poorly understood nature of formaldehyde induced cross-linking in living cells, this problem is also largely insuperable.

The third problem arises when ChIP-seq is used to compare the occupancy of different cell states. Differences in ChIP-seq profiles from different samples can reveal changes between cell states in the genomic distribution of proteins but not in occupancy per se. For example, if occupancy were reduced or increased at all loci throughout the genome in a similar manner, then conventional ChIP-seq would not reveal any change. This fact has been widely ignored in the field and has been source of much confusion. Nevertheless, measuring changes in the occupancy of chromosomal proteins at specific loci within the genome is crucial for evaluating their function. Hitherto, this has been achieved using quantitative PCR to measure DNAs that have been crosslinked to specific proteins. However, this technique can only sample a minute fraction of the genome and as a consequence it cannot distinguish whether a reduction at a given locus is caused by reduced loading throughout the genome or merely by a change in distribution. What is required is a method that measures differences in occupancy between states as well as between different genomic loci in a manner that encompasses the entire genome. This goal, namely to measure changes in any protein's occupancy/activity throughout all chromosomes of a cell, we refer to as biological chromodynamics.

Though the problem of how to measure differences in occupancies between samples is a serious one, it fortunately has a simple solution. We show here that the problem of how to ‘calibrate’ ChIP-seq profiles can be solved by mixing experimental samples with a single (calibration) sample from an organism whose sequences can be distinguished and whose physiology is sufficiently similar that the processes of fixation, DNA sonication, and IP work in a similar manner to experimental samples. The only condition required for this technique to work is that the calibration and experimental genomes express proteins containing the same epitope used for immunoprecipitation.

We describe here the creation of a strain of *Candida glabrata* that expresses a PK-tagged cohesin subunit that can be used to calibrate ChIP-seq profiles of cohesin tagged with the same epitope from *Saccharomyces cerevisiae*. While this work was in preparation, a similar concept has been described using antibodies specific for Pol II (4) or histone modifications (5). Our application differs from these in a number of respects. First, we present a different albeit very simple mathematical formulation explaining why the method works. Second, we describe a method in which the internal control is added at an earlier step, namely by mixing live cells, a feature that controls for possible variations in fixation among different experimental samples. Third, we demonstrate for the first time that an internal control permits occupancy measurements that are linearly proportional to actual occupancy over all ranges of values, which is a *sine qua non* for any truly quantitative method. Fourth, by tagging proteins in experimental (*S. cerevisiae*) and calibration (*C. glabrata*) genomes with the same epitopes, we describe a method that can be used for any protein not merely for those with highly conserved epitopes, which are quite rare. Fifth, we actually use the method to document changes in occupancy in response to mutations and as wild type cells undergo changes in their physiological state. Last but not least, by comparing the calibrated ChIP-seq profiles of tagged and untagged strains, we determine for the first time the fraction of reads at any locus that derive from genuine *in vivo* occupancy as opposed to the background noise that compromises all published data sets. A reliable method for distinguishing signals from noise has never previously been described. Because of this feature, we are able to evaluate reads that are not within defined peaks. In the case of cohesin as well as many other chromosomal proteins that are not sequence-specific DNA binding proteins, the majority of reads are not found in defined peaks. These data points have frequently been ignored on the premise that they constitute a background due to the IP not being entirely specific. Our technique reveals that this is not necessarily the case and that reads between peaks must therefore be taken more seriously.

## MATERIALS AND METHODS

### Logic of using a calibration genome

Conventional ChIP-seq reveals information about the distribution of occupancies of chromosomal proteins across the genome but because of the complexity of the procedure and the impossibility (see below) of making each step fully quantitative, it cannot be used to compare occupancies between two different samples. Our goal is to convert conventional ChIP-seq genome distributions to ones that provide information also about differences in occupancy between experimental samples. The principle behind the method is the use of a second ‘calibration’ genome to provide an internal control for the experimental genome. All that is required is that cells with the experimental and calibration genomes possess the same epitope that will be immunoprecipitated, that cells with the calibration genome are added (preferably prior to fixation with formaldehyde) to the cells with the experimental genome, and that the different aliquots of cells with the calibration genome added to different samples of cells with the experimental genome are in exactly the same state, in this case asynchronous logarithmic phase growth. We note that doing this with cells from multicellular organisms may not be feasible using the protocol described here and solving this limitation is a challenge for the future. Moreover, our calibration method does not provide any insight into cell to cell variation.

Let *N*_X_ = number of cells from experimental genome X and *N*_C_ = number of cells from calibration genome C that are mixed either before or after fixation but prior to all subsequent steps associated with the ChIP-seq protocol. Let the number of reads (within PK tag immunoprecipitates) assigned to experimental genome X and calibration genome C = *IP_X_* and *IP_C_*, respectively. Let *O_X_* = the occupancy (averaged over the entire genome) on the experimental genome X of PK-tagged protein *Z_X_*. The following may be helpful in clarifying what *O_X_* means. At each base pair along the genome, there is a certain probability that protein *Z_X_* is present. *O_X_* is the average among all base pairs along the genome of these probabilities. Meanwhile, let *E_X_* = the combined efficiency (associated with cells with genome X) of cross-linking, cell breakage, DNA sonication, immunoprecipitation (for a given number of epitopes), persistence of DNAs within the immunoprecipitates during the washing procedure, release from the immunoprecipitation beads, de-crosslinking, subsequent DNA purification, library construction, library amplification by PCR, and finally the sequencing reaction. *E_X_* is therefore a measure of the efficiency of the entire procedure for the experimental cells. Note that due to the complexity of the procedures, few if any of the steps combined in *E_X_* will be identical between samples. Thus *E_X_* will undoubtedly vary considerably between samples. Moreover, there is little prospect that it could ever be standardized. Let *O_C_* = the occupancy (averaged over the entire genome) on the calibration genome C of PK-tagged protein *Z_C_* and *E_C_* be the combined efficiency of the procedure (as defined above) for cells with the calibration genome C. Accordingly, *IP_X_* = *N_X_O_X_E_X_* and *IP_C_* = *N_C_O_C_E_C_*. The fraction of reads from the experimental genome X within cross-linked samples that have been immunoprecipitated = *F_X_* = *IP_X_*/(*IP_X_* + *IP_C_*). Because *IP_X_* = *N_X_O_X_E_X_* and *IP_C_* = *N_C_O_C_E_C_*, then *F_X_* = *N_X_O_X_E_X_*/(*N_X_O_X_E_X_* + *N_C_O_C_E_C_*). Reorganizing this equation, *O_X_E_X_*/*O_C_E_C_* = *N_C_F_X_*/(*N_X_*(1 − *F_X_*)). Note that the fraction of reads assigned to the calibration genome *F_C_* = 1 − *F_X_*.

The key insight behind our calibration method is the following. Though *E_X_* and *E_C_* will both vary between two different cell samples, the variations will be identical because cells with genome X and C are mixed in the same flask at the beginning of the experiment. In other words, *E_X_*/*E_C_* will be a constant. Note also that because cells with the calibration genome are grown under constant conditions for any given experiment in which cells with the experimental genome are varied, *O_C_* will also not vary. For simplicity, let us rename the invariant *O_C_E_C_* /*E_X_* ratio as the constant **α**. Using this nomenclature, occupancy associated with the mixture of calibration and experimental cells associated with sample **i** of cells with the experimental genome, *O_X**i**_* = **α***N_C**i**_F_X**i**_*/(*N_X**i**_*(1 − *F_X**i**_*)). In other words, *O_X**i**_* is directly proportional to *N_C**i**_F_X**i**_*/(*N_X**i**_*(1 − *F_X**i**_*)), which is therefore a measure of the occupancy of protein *Z_X_* on genome X from sample **i** relative to *Z_C_* on genome C under standard (and invariant) conditions. We call this term the occupancy ratio of sample **i** or *OR**_i_***. In conclusion, *O_X**i**_* = **α***OR**_i_***, where *OR**_i_*** = *N_C**i**_F_X**i**_*/(*N_X**i**_*(1 − *F_X**i**_*)). We do not know the value of **α**, but this does not matter as we can assume that it is an invariant parameter.

Having assigned reads from IP samples to experimental and calibration genomes, reads from the experimental genome are plotted as reads per million at each base pair along the genome. When plotting the sequence data in this manner, namely reads at each base pair along the genome as a ratio of the number reads assigned to that base pair divided by the total number of reads, one obtains a measure of the distribution of the protein across the genome but no information about differences in occupancy between two samples. This is the key limitation of conventional ChIP-seq that hitherto has not been fully appreciated. To obtain distributions that reflect occupancy (relative to other samples), one merely has to plot the product of the distribution and the occupancy ratio (*OR**_i_***) for that sample. To do this, each value (reads per million) associated with each base pair within the genome is merely multiplied by the *OR**_i_*** calculated for that sample *i* to obtain distributions whose values at different loci or base pairs (whether within peaks or troughs) now represent a quantitative measure of the relative occupancy of the protein at that locus among different samples. Note that these are not absolute occupancies but merely ones that can be compared quantitatively between samples. By this means, one obtains occupancies at all loci within the genome that can be compared quantitatively to occupancies at the same set of loci in other samples of cells with the experimental genome.

We refer to this process as ‘calibrating the distributions’ and the process as ‘calibrated ChIP-seq’. Calculating *OR**_i_*** is the key to the calibration process. The key point is that because *OR**_i_*** = *N_C**i**_F_X**i**_*/(*N_X**i**_*(1 − *F_X**i**_*)), all we need to know is *N_C**i**_*/ *N_X**i**_* and *F_X**i**_*. *N_C_* and *N_X_* can be measured by cell counting at the time of mixing cell cultures. Simpler still, under steady state conditions (i.e. in exponentially growing asynchronous cultures) *N_C_*/*N_X_* is given by *W_C_*/*W_X_*, where *W_C_* and *W_X_* are the reads assigned to genomes C and X in aliquots from our samples that have not been immunoprecipitated (whole cell extracts or WCE). Meanwhile, *F_X_* = *IP_X_*/(*IP_X_* + *IP_C_*), where *IP_X_* and *IP_C_* are derived directly from the sequence data. As a consequence, *OR**_i_*** = *W_C**i**_IP_X**i**_* /*W_X**i**_ IP_C**i**_*.

In summary, by adding cells with the calibration genome to different samples of cells with the experimental genome, it is possible to calculate *OR**_i_*** for experimental sample *i* from the number of reads assigned to calibration (*W_C**i**_*) and experimental (*W_X__**i**_* ) genomes among sequences derived from aliquots removed prior to immunoprecipitation and the number of reads assigned to calibration (*IP_C**i**_* ) and experimental (*IP_X**i**_* ) genomes among sequences derived from aliquots that have been immunoprecipitated. The calibration process is therefore incredibly simple and transforms the nature of the data that one can obtain from ChIP-seq. Though the principle of using a calibration genome has been reported in two previous papers (4,5), neither of these explain in the same simple mathematical terms the logic of the procedure as outlined above. We note also that neither paper demonstrated that calibration genomes can actually be utilized in the manner described above to produce fully quantitative comparisons between experimental samples, as we show in this paper. To minimize variation caused by differences in fixation between samples, we recommend that calibration be performed by mixing calibration and experimental cells prior to treating the mixture with formaldehyde. Though this is clearly the optimum strategy, in some of our experiments we used pre-fixed calibration cells, which were added to samples of fixed experimental cells.

### Yeast strains, media and culture

The budding yeast, *S. cerevisiae*, the source of our ‘experimental’ samples, was derived from W303 strain background while the ‘calibration’ yeast, *C. glabrata*, was obtained from the National Collection of Yeast Cultures (NCYC No 388). Both yeast strains were grown in YPD (1% yeast extract, 2% peptone and 2% glucose) using standard culture methods. Unless otherwise stated, all cultures were grown at 25°C. To generate a *C. glabrata* strain whose endogenous *SCC1* locus expressed Scc1 protein tagged with PK epitopes at its C-terminus, we constructed a homologue integration cassette containing a pair of 500bp DNA sequences separated by a restrictive site EcoRV. These sequences were homologous to the region immediately upstream and downstream of the *C. glabrata SCC1* stop codon respectively. A DNA fragment containing a PK epitope tag and a NatMX6 module was amplified by PCR and subcloned into the EcoRV site of the homologue integration cassette. The final PK9-NatMX integration cassette carrying the 500 bp long flanking regions was transformed into a *C. glabrata* strain to generate the strain K23308 encoding nine repeats of the PK epitope tag at the carboxyl-terminal ends of *C. glabrata SCC1*, using a modified LiOAc transformation method as previously described (6). The expression of PK epitope-tagged Scc1 protein in *S. cerevisiae* and *C. glabrata* strains was confirmed by immunoblotting analysis (Supplementary Figure S2).

To arrest S *cerevisiae* in G1 phase, α-Factor mating pheromone was added to a final concentration of 5μg/ml and cells were shaken at 25°C for 2.5 h until most had projected shmoos. To release cells, α-Factor was removed by filtration and cells were washed with equal volume of YPD. Subsequently, the cells were re-suspended into YPD media and shaken at 25°C. The samples were taken every 15 min for 2 h.

### Chromatin-immunoprecipitation and Deep sequencing

Yeast cells were grown in YPD to reach a density of 0.3–0.6 OD_600_. To crosslink cells, 45 ml of yeast culture was mixed with 4.2 ml of fixation solution (50mM Tris–HCl pH 8.0, 100 mM NaCl, 0.5 mM EGTA, 1 mM EDTA, 30% Formaldehyde) and incubated at 18°C for 30 min. The crosslinking reaction was quenched by incubating with 2 ml of 2.5 M glycine for 5 min. Fixed cells were harvested, washed with ice-cold PBS and re-suspended in 1 ml of ChIP lysis buffer (50 mM Hepes–KOH pH 8.0, 140 mM NaCl, 1 mM EDTA, 1% Triton X-100, 0.1% sodium deoxycholate, 1 mM PMSF, Roche protease inhibitor). For each ChIP, 10 OD_600_ units of *S. cerevisiae* cells mixed with 5 OD_600_ units of *C. glabrata* cells were pelleted and re-suspended in 0.3 ml of ChIP lysis buffer. Cells were mixed with glass beads and disrupted by FastPrep^®^-24 (MP Biomedicals, USA). The entire lysis was collected and sonicated at 4°C for 40 min using a Bioruptor (Diagenode, Belgium). The cell debris were removed by centrifugation and supernatants containing sheared chromatin with a size range from 100 to 800 bp were adjusted to a final volume of 1ml with ChIP lysis buffer. Extracts were pre-cleared for 1 h at 4°C with 30 μl of Protein G Dynabeads (Invitrogen). 80 μl of supernatant was taken as whole cell extract (W) and stored at −80°C. Five μg of anti-PK antibody (Bio-Rad) and 50 μl of Protein G Dynal beads (Invitrogen) was used for immunoprecipitation (6 h-overnight, rotation at 4°C). The beads were subsequently washed for 5 min with the following buffers: 2× ChIP lysis buffer; 3x ChIP high-salt lysis buffer (50 mM Hepes–KOH pH 8.0, 500 mM NaCl, 1mM EDTA, 1% Triton X-100, 0.1% sodium deoxycholate, 1 mM PMSF); 2× ChIP wash buffer (10 mM Tris–HCl pH 8.0, 0.25 M LiCl, 0.5% NP-40, 0.5% sodium deoxycholate, 1 mM EDTA, 1 mM PMSF) and 1x TE buffer (10 mM Tris–HCl pH 8.0, 1 mM EDTA, 50 mM NaCl). The immunoprecipitated chromatin was eluted by incubation of beads with 120 μl of TES buffer (50 mM Tris–HCl pH 8.0; 10 mM EDTA; 1% SDS) at 65°C for 15 min. The supernatants were collected and termed the IP sample. The whole cell extract sample (W) was mixed with 40 μl of TES3 buffer (50 mM Tris–HCl pH 8.0; 10 mM EDTA; 3% SDS). Both samples were de-crosslinked at 65°C overnight. RNA was degraded by incubating with 2-μl of RNAse A (10 mg/ml, Roche) at 37°C for 1 h and protein was subsequently removed by incubation with 10 μl of Proteinase K (18 mg/ml, Roche) at 65°C for 2 h. DNA was purified using ChIP DNA Clean & Concentrator kit (Zymo Research, USA).

For each sample, a sequencing library was constructed using NEBNext^®^ Fast DNA Library Prep Set for Ion Torrent™ Kit (NEB, USA). Briefly, DNA fragments (10–100 ng) were converted to blunt ends by end repair and ligated with Ion Xpress™ Barcode Adapters. The resulting DNA fragments with a size of 300bp were selected using E-Gel^®^ SizeSelect™ Agarose Gels 2% (Life Technologies, USA) and amplified by PCR using 6–8 cycles. DNA concentration was determined by qPCR and adjusted to 300 pM. Eight to twelve libraries with different barcodes were pooled together and loaded onto The Ion PI™ Chip v2 BC using the Ion Chef™ Instrument (Life Technologies, USA). Library sequencing was carried out on the Ion Torrent Proton. Typically, sequencing of one ChIP library generates 6–10 million reads with average length of 190 bp, covering the yeast genome more than 100 times. The sequencing data generated in this study has been deposited in GEO with the accession number GSE69907. Sequencing of samples from a given experiment/comparison was usually performed on a single sequencing run. In cases, where all samples could not be accommodated in a single run, then we made sure that all IP samples were sequenced in a single run and all WCE samples in another single run.

### Data processing and reads alignment

All data processing was carried out on the Galaxy platform (7). The quality of the raw sequence data was examined using FastQC (Galaxy tool version 1.0.0) and trimming of reads was carried out using ‘trim sequences’ (Galaxy tool version 1.0.0). Trimming usually required removing the first 10 bp and bases after the 200th. Although trimming more bases from the end of the reads may be required to remove Kmers and ensure the per base sequence content is equal across the reads. To minimize misalignment between two genomes, any reads smaller than 50 bp were removed using ‘Filter FASTQ’ (Galaxy tool version 1.0.0) (minimum size: 50, maximum size: 0, minimum quality: 0, maximum quality: 0, maximum number of bases allowed outside of quality range: 0, paired end data: False). The final reads were aligned to the *S. cerevisiae* genome (sacCer3, SGD) or *C. glabrata* genome (CBS138, genolevures) using Bowtie2 (Galaxy tool version 0.2) with the default (–sensitive) parameters (8) (Mate paired: Single-end, write unaligned reads to separate file: True, Reference genome: SacCer3 or CanGla, Specify read group: false, parameter settings: full parameter list, type of alignment: end to end, preset option: sensitive, disallow gaps within *n*-postitions of read: 4, Trim *n*-bases from 5′ of each read: 0, Trim *n*-bases from 3′ of each read: 0, skip the first n-reads: 0, number of reads to be aligned: 0, strand directions: both, log mapping time: false).

To generate an alignment in which all the sequences exclusively align to the *S. cerevisiae* genome, the whole reads were first aligned to *C. glabrata* genome and the unaligned reads were retrieved as a separate Fastq file. Subsequently, these unaligned reads were re-aligned to *S. cerevisiae* genome and the resulting aligned BAM file therefore contained reads that were unique to *Saccharomyces cerevisiae*. A same strategy was used to generate alignments unique to *C. glabrata*.

To generate data sets of reads that align to both the *S. cerevisiae* and the *C. glabrata* genomes, all the sequences were first aligned to *S. cerevisiae* genome and unaligned sequences were removed using ‘Filter SAM or BAM’ (Galaxy tool version 1.1.0) with a setting of Skip alignments with any of these flag bits set as ‘The read is unmapped’ (9). The obtained BAM file was converted to a Fastq file. The remaining sequences were re-aligned to the *C. glabrata* genome and unaligned sequences were again removed. The final converted Fastq file therefore contains those reads that align to both genomes.

### Visualization of ChIP-seq data

All the aligned sequencing reads were visualised in the IGB browser (10). First, the alignment BAM file was converted to BigWig format using the ‘BAM to BigWig’ (Galaxy tool version 0.1.0) function on the Galaxy platform. The genome-wide ChIP signal can then be visualised in the IGB browser on a basis of per million reads (conventional ChIP-seq). To obtain a calibrated version, the browser track with the conventional ChIP-seq profile was multiplied by the occupancy ratio (OR) corresponding to that sample.

To calculate the average occupancy at each base pair around all 16 centromeres, the BAM file containing reads uniquely aligned to *S.cerevisiae* were separated into separate files for each chromosome using the ‘Filter SAM or BAM’ function on the galaxy platform. A pileup for each chromosome was then obtained using samtools Mpileup (Galaxy tool version 0.0.1) (source for reference list: locally cached, reference genome: SacCer3, genotype likelihood computation: false, advanced options: basic), which was then edited to assign unrepresented chromosome positions to 0. The pileup was then filtered to obtain the number of reads at each base pair within 15 kb or more intervals either side of the centromeric CDEIII elements within each chromosome. The number of reads at successive bases as one moves away from CDEIII could then be averaged across all 16 chromosomes and calibrated. In yeast, the Cbf3 factor initiates kinetochore assembly by binding to highly conserved CDEIII sequences found at centromeres of all 16 chromosomes.

## RESULTS

### A method for calibrating ChIP-seq

Conventional ChIP-seq allows one to compare occupancies of proteins at different positions within a genome but not occupancies at a given position between samples. Given the importance of ChIP-seq to chromosome biology, there is a dire need for a process that can calibrate different ChIP-seq profiles so that that they represent not merely genomic distributions but also differences in occupancy between samples of experimental cells in different states. To do this, it is necessary to compare in a fully quantitative manner the efficiency with which DNA sequences are immunoprecipitated from different experimental samples. The problem is that the entire ChIP process is inherently complex and involves numerous steps, some which cannot be performed in a totally reliable (i.e. quantitative) manner. Thus samples will vary to a greater or lesser extent in the efficiency of cross-linking, cell breakage, DNA sonication, immunoprecipitation (for a given number of epitopes), persistence of DNAs within the immunoprecipitates during the washing procedure, release from the immunoprecipitation beads, de-crosslinking, subsequent DNA purification, library construction, library amplification by PCR, and finally the sequencing reaction. Even if several of these steps are performed in a routine and reliable manner, variations in just one will compromise the ability to obtain quantitative data.

A solution to this problem is to introduce a marking system that allows one to measure the actual variation between samples of each step in the procedure. Fortunately, this is simpler to do than might be imagined. One merely has to add to the experimental cells prior to the start of the procedure a second set of living cells (the calibration cells) whose physiology is similar to that of the experimental cells but whose sequences are distinguishable. If the calibration cells express proteins with the same epitope used for immunoprecipitation and if the calibration cells added to different samples of experimental cells are, unlike the experimental cells, always in the same physiological state and therefore can be assumed to have identical occupancies at all positions within their genome of the protein sharing the epitope with that expressed by the experimental cells, then one can compare the genomic occupancies of the protein in question between calibration and experimental cells.

As outlined in detail in materials and methods, the occupancy (*O_X**i**_* ) averaged over the entire experimental genome (X) of our epitope tagged protein in sample *i* = *αN*_C*i*_*F*_X**i**_ /(*N_X**i**_* (1 − *F_X**i**_* )), where *α* is a constant, *N_C**i**_* is the number of calibration cells added to sample *i* of experimental cells, *N*_X**i**_ the number of experimental cells in sample *i* mixed with these calibration cells, and *F*_X*i*_ the fraction of reads assigned to the experimental genome X after sequencing aliquots that have been immunoprecipitated from experimental-calibration cell mixture sample *i*. We call *N*_C*i*_*F*_X*i*_ /(*N*_X*i*_(1 − *F*_X*i*_)) the occupancy ratio or *OR_i_*. Because it is directly proportional to *O_Xi_* and can be calculated using *N*_C*i*_ /*N*_X*i*_ and *F*_X*i*_ , it provides the metric to calibrate our ChIP-seq distributions so that they provide information about occupancy. In actual terms, OR measures the probability of detecting a protein on the experimental genome using ChIP-seq compared with the probability of detecting the same or a different protein tagged with the same epitope on the calibration genome. Using the *OR**_i_*** of each combination i of experimental and (invariant) calibration cells, it is possible to convert the distributions revealed by the ChIP-seq profiles into a format that allows quantitative comparisons between samples not merely in the distribution across the genome but also in occupancy at any position in the genome.

ChIP-seq profiles from the experimental genome are first normalized to total reads associated with each base pair for each million total sequences (the density data) and then calibrated by multiplying the density data associated with each base by the *OR**_i_*** of that sample. It is important to note that we ignore all reads that either aligned to both genomes or for some reason aligned to neither. To avoid having to measure cell number, we determined *N*_C*i*_ /*N*_X*i*_ by calculating *W*_C*i*_ /*W*_X*i*_ where *W*_C*i*_ and *W*_X*i*_ are the number of reads unique to the calibration and experimental genomes respectively in samples obtained from whole cell extracts (i.e. aliquots from the same mixture of calibration and experimental cells that had been cross-linked and sonicated but not subjected to the IP protocol). This method is not obligatory but avoids any possibility of making mistakes in measuring cell numbers and ensures that *OR**_i_*** is calculated on a per genome (or per chromatid) basis, which is more relevant than a per cell basis. Thus, *OR_i_* = *W_Ci_* /(*W*_*Xi*_ (1 − *F*_X*i*_ )) = *W_Ci_ IP_Xi_* /*W*_X*i*_*IP_Ci_* , where *IP_Xi_* and *IP_Ci_* are the number of reads assigned to experimental and calibration genomes in immunoprecipitated aliquots (from mixture *i*). The detailed mathematical logic of this principle is explained in materials and methods.

Because very different types of biological material might respond differently to variations in sample preparation, it is ideal that the calibration genome be from an organism that is as closely related as possible in its physiology to the experimental genome and yet be sufficiently different to assign most sequences to either calibration or experimental genomes. Because our experimental genome was the budding yeast *S. cerevisiae*, we chose as our calibration genome that of *C. glabrata*. For obvious reasons, our calibration method will not work well if significant parts of the genome cannot be assigned correctly, which is why we chose for calibration a yeast whose sequence was sufficiently different. As described below, this condition is satisfied for most sequences in the case of *S. cerevisiae* and *C. glabrata* but it is not true for sequences encoding rRNAs. In summary, then, two or more experimental *S. cerevisiae* cell samples are mixed in each case with a defined amount of calibration *C. glabrata* cells, the cell mixtures are cross-linked, broken, and sonicated, at which point one aliquot is immunoprecipated (IP) and another not (W), and both then processed further independently for DNA sequencing (Supplementary Figure S1). We next calculate the number of reads assigned to calibration (C) and experimental (X) genomes within the IP and W samples and thereby calculate the OR for each mixture (*W*_C_*IP_X_* /*W*_X_*IP_C_*), which is then used to calibrate the ChIP-seq distribution, which is plotted on a linear scale. If the calibration works as predicted, the height of each point on the calibrated distribution should be directly proportional to occupancy, allowing comparisons of occupancy not only within the *S. cerevisiae* genome but also between genomes from different *S. cerevisiae* samples.

### Validating the calibration method

To validate our method, we applied it to the cohesin complex whose primary function is to mediate sister chromatid cohesion. In *S. cerevisiae*, most if not all of cohesin's Scc1 subunit is cleaved by separase at the metaphase to anaphase transition, which triggers immediate release of the complex from chromosomes (11). They are not re-generated until late G1 of the subsequent cell cycle when there is a burst of new synthesis. Cohesin is therefore an example of a complex whose association with chromosomes is tightly cell cycle regulated, a process that one would like to measure using calibrated ChIP-seq. To do this, we created *Saccharomyces cerevisiae* (K14601) and *C. glabrata* (K23308) strains whose Scc1 subunits were tagged at their C-termini with nine tandem repeats of the PK epitope (Supplementary Figure S2), which enabled us to immunoprecipitate the *S.cerevisiae* and *C. glabrata* proteins in a single ChIP reaction. We made no attempt to address whether their cross-linking to DNA or immunoprecipitation occurred with identical efficiencies as our method does not depend on this being true. However, given the relatedness of the two organisms, this is likely to be the case.

**Figure 1.**
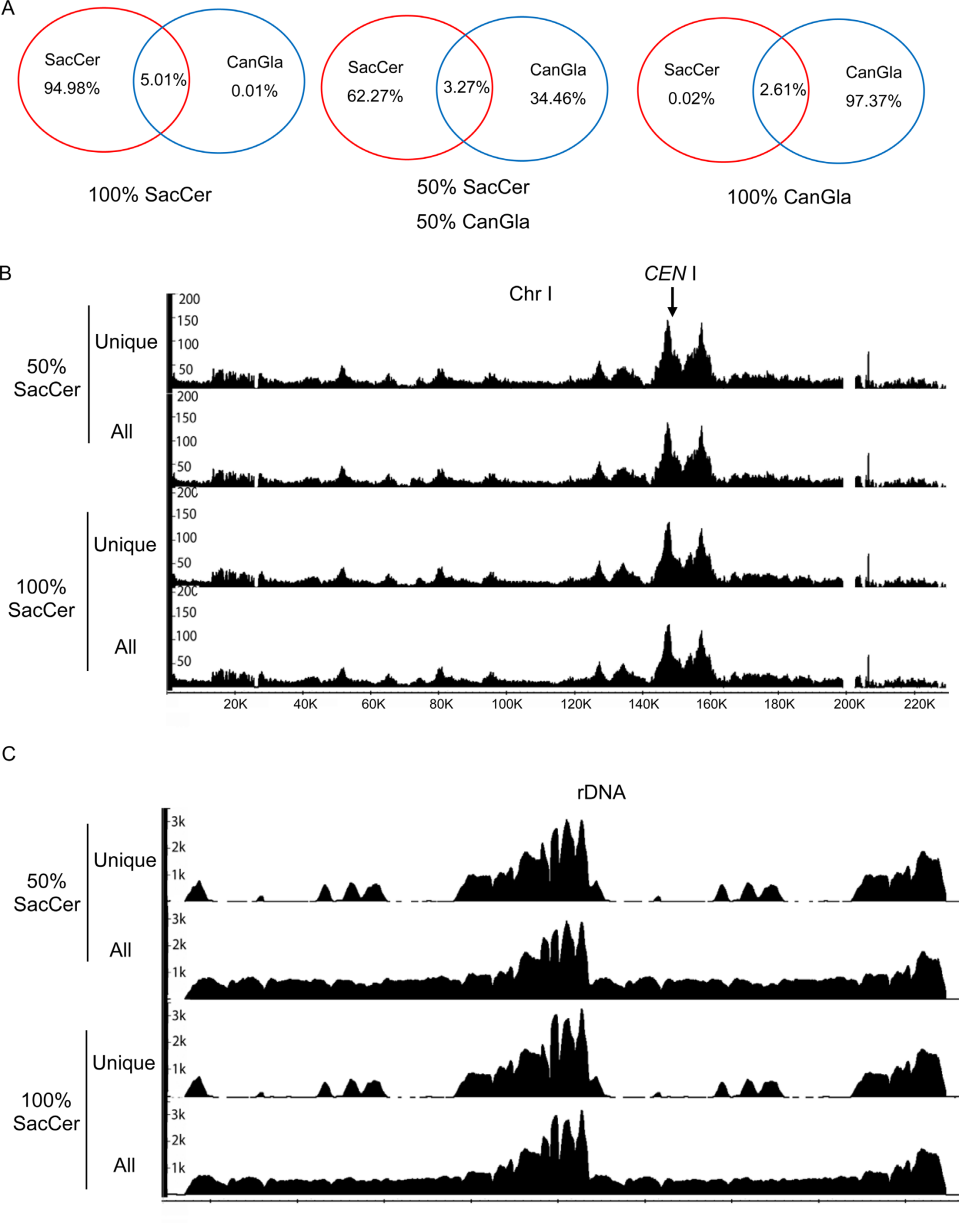
ChIP-seq profiles are unaffected by reference cells. Crude extracts prepared from exponentially grown *S. cerevisiae* cells (K14601, *MATa*, Scc1PK9::KanMX), *C. glabrata* (K23308, *MATa*, Scc1PK9::NatMX), or a mixture were processed for ChIP-seq. (**A**) All sequences from whole cell extracts (W) were aligned to the experimental (*S.cerevisiae*) and reference genomes (*C. glabrata*) or both. The numbers in the left (red) or right (blue) circles indicate the percentage of reads that align uniquely to *S. cerevisiae* or *C. glabrata* genomes. Those in the intersection indicate the percentage of reads that align to both. ChIP-seq distributions of SacCer_Scc1 on chromosome I (**B**) or rDNA region (**C**), aligning either all reads or only those unique to *S. cerevisiae*, from pure *S. cerevisiae* or mixed cultures respectively. The Y-axis indicates the numbers of reads covering every base pair and the X-axis indicates position of every base pair adopted from SGD (http://www.yeastgenome.org).

A key issue is whether sequences obtained after chromatin immunoprecipitation could be assigned with high accuracy to their parental genomes. To evaluate the fraction of reads that aligned to both genomes, we performed Scc1-PK ChIP-seq from a pure culture of *S.cerevisiae*, from a pure culture of *C. glabrata*, and an equal mixture of the two strains. *S.cerevisiae* and *C. glabrata* were grown separately to exponential phase in YPD at 25°C and both cultures diluted to the same optical density. The two pure cultures and a mixture containing equal quantities were fixed with formaldehyde and processed as if for ChIP-seq. In the absence of immunoprecipitation, about 5% of sequences from the *S. cerevisiae* culture aligned to the *C. glabrata* genome while 2.6% of sequences from the *C. glabrata* culture aligned to *S. cerevisiae* genome (Figure 1A). Importantly, only 3.27% of sequences from the mixed culture aligned to both genomes. Most of these (80%) were sequences from rDNA loci and the rest were regions that encode parts of highly conserved proteins involved in translation and metabolism. Importantly, elimination of sequences that aligned to both genomes has very little effect on Scc1's ChIP-seq profile throughout most of the *S. cerevisiae* genome (Figure 1B). In contrast, it has a major effect on the profile within rDNA, with large swathes of these loci being depleted for assigned sequences (Figure 1C). Because rDNA occupies 10% of the total genome in *S. cerevisiae*, sequences from this locus account for most of the *S.cerevisiae* sequences that can also be aligned to *C. glabrata* (Supplementary Figure S3). These findings show that *C. glabrata* should be suitable for calibrating ChIP-seq profiles for most of the *S.cerevisiae* genome but not for rDNAs. This was confirmed by a scatter diagram of values obtained using all sequences plotted against those unique to *S. cerevisiae*, which revealed a correlation coefficient of 0.997 (Supplementary Figure S3C).

**Figure 2.**
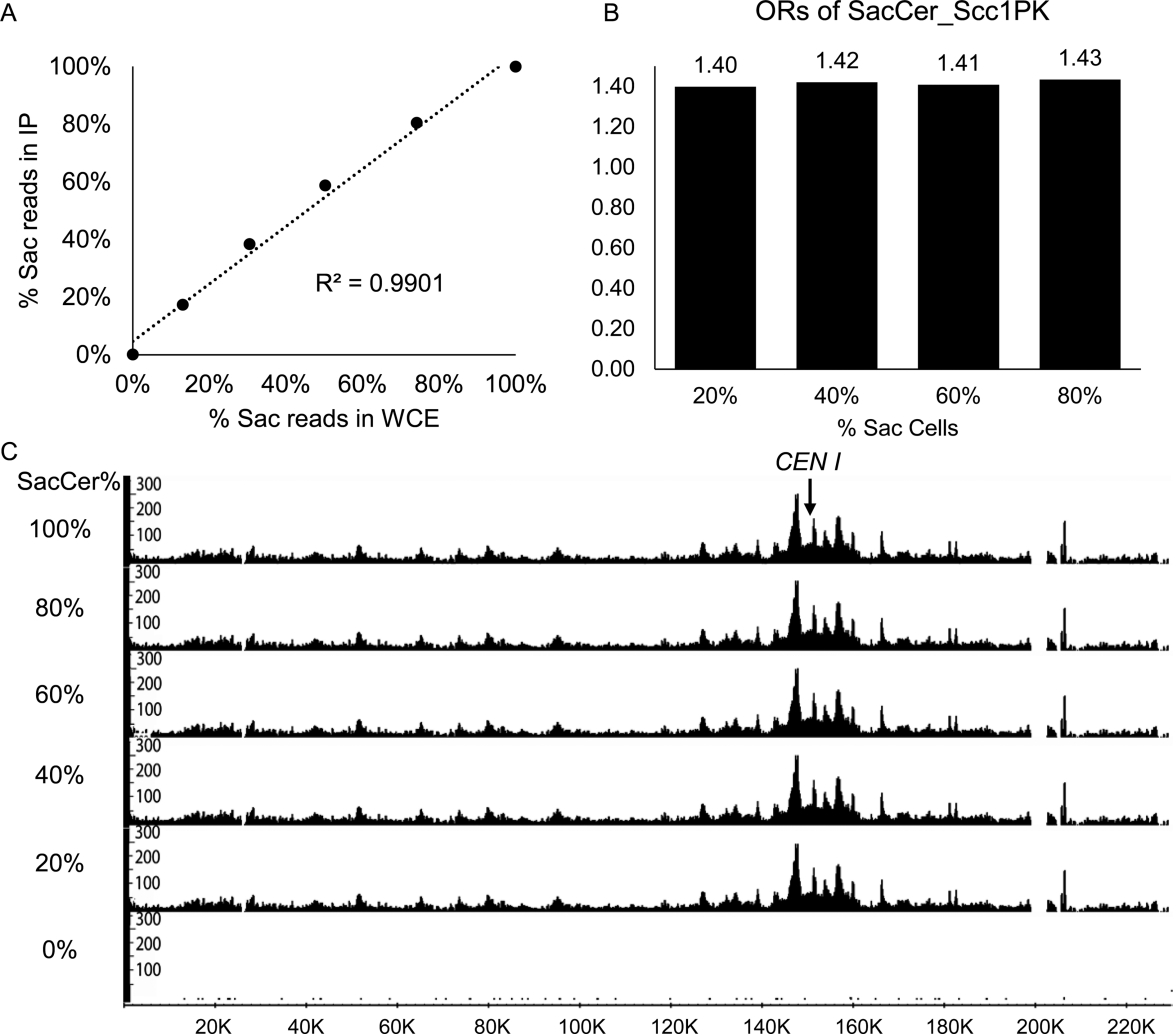
Occupancy ratio (OR) is unaffected by the amount of reference cells. ChIP-seq experiments were carried out from exponentially growing *S. cerevisiae* cells mixed with exponentially growing *C. glabrata* cells in the indicated ratios. Only unique reads to either genome were used for alignment. (**A**) Correlation between percentages of reads aligning to *S. cerevisiae* genome from IP and whole cell extract (W) samples. (**B**) ORs of mixtures with the indicated ratios. (**C**) ChIP-seq distributions of SacCer_Scc1 on chromosome I from mixtures with different ratios of *S. cerevisiae* and *C. glabrata* cells. The Y-axis indicates the numbers of reads covering every base pair and the X-axis indicates position of every base pair adopting from SGD (http://www.yeastgenome.org).

If mixing cells from different species is to be used to calibrate ChIP-seq profiles, then it is essential to ascertain that adding *C. glabrata* cells to *S. cerevisiae* cultures does not influence the OR of DNA sequences unique to *S. cerevisiae*. To address this, we made a series of cultures containing varying ratios of exponentially growing *S. cerevisiae* and *C. glabrata* cells (0:100, 20:80, 40:60, 60:40, 80:20 and 100:0 volume ratios) and collected data sets from samples before (whole cell extract or W) and after immunoprecipitation (IP). Crucially, the percentage of reads unique to *S. cerevisiae* in IP samples increased in a linear fashion (*R*^2^ = 0.9901) when plotted against the equivalent percentage from whole cell extracts (W) (Figure 2A), which reveals the actual ratios of chromatids from the two species. We note that neither of the two previous attempts to use internal or reference genomes to quantitative ChIP-seq profiles (4,5) demonstrated this linear relationship, without which no calibration method can be used reliably.

Crucially, ORs calculated from the four different data sets including both types of cells were almost identical, with a figure around 1.4 (Figure 2B), suggesting that it is slightly easier to IP *S. cerevisiae* DNA with Scc1-PK than *C. glabrata* DNA. Whether this difference is due to genuine differences in occupancy or cross-linking efficiency is immaterial to the method. As expected given the above result, varying the fraction of *S.cerevisiae* cells from 20 to 80% had little or no effect on Scc1's *S. cerevisiae* genomic profile either when plotted on the reads-per-million basis (Figure 2C) or when calibrated by multiplying by the corresponding OR (Supplementary Figure S4A). This was confirmed by a scatter diagram of the ChIP signals of every base pair from the whole genome obtained using all sequences plotted against those unique to *S. cerevisiae*, which revealed a correlation coefficient of 0.997 (Supplementary Figure S3C).

As in *S. cerevisiae*, cohesin in *C. glabrata* is enriched in the region surrounding centromeres and concentrated within intergenic regions where there is convergent transcription (Supplementary Figure S5). This demonstration that OR measurements remain invariant despite extreme variations in the ratio of experimental and calibrations reads is crucial to our calibration method. For example, it is vital that the process we use to generate and assign reads detects infrequent reads from the experimental genome amongst a majority of reads from the calibration genome (as will be the case when occupancy of the experimental genome is low) with the same efficiency as when reads from the experimental genome are in the majority, as may be the case when occupancy of the experimental genome is high.

### Measuring cohesin's cell cycle chromodynamics using calibrated ChIP-seq

**Figure 3.**
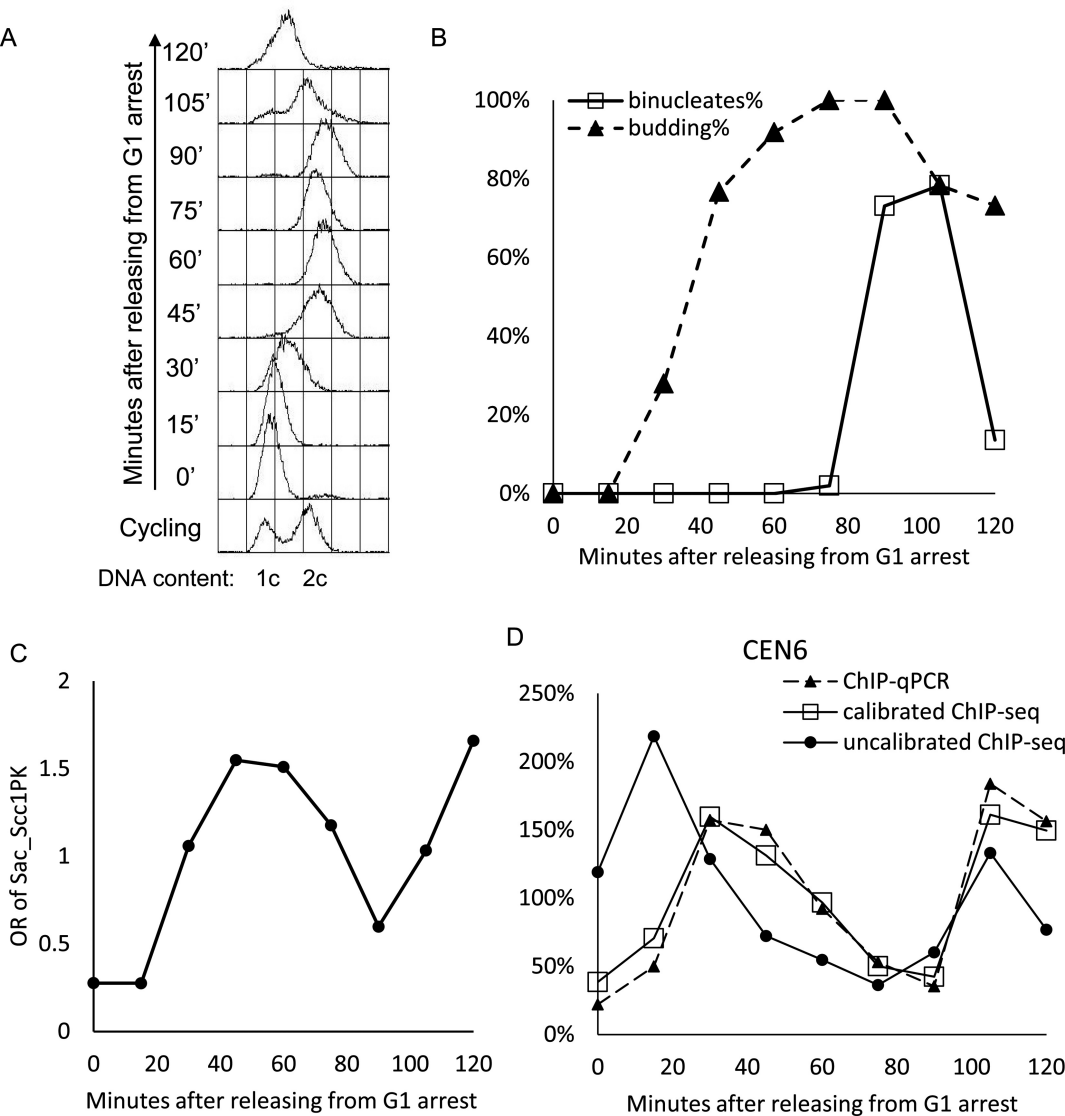
Using calibrated ChIP-seq to measure cohesin's cell cycle dependent association with the *S. cerevisiae* genome. Exponential phase *S. cerevisiae* cells growing at 25°C were arrested in G1 by treatment with α-factor pheromone for 150 min. and then transferred by filtration to fresh medium lacking the pheromone. This triggered their synchronous passage through the cell cycle. At the indicated time points, samples were removed from the synchronous *S.cerevisiae* culture to measure DNA content by FACS (**A**), the fraction of cells with buds (budding index) (**B**), the fraction of binucleate cells (B), and OR values for each time point (**C**). In the case of the latter, *S.cerevisiae* cell samples were first fixed with formaldehyde and only subsequently mixed with pre-fixed *C. glabrata* cells, a protocol that made it simpler to sample continuously for up to 120 min. from the *S. cerevisiae* synchronous culture. Occupancy ratio (OR) values for PK-tagged Scc1 were calculated for each time point using reads unique to *S. cerevisiae* and *C. glabrata* cells in IP and whole cell extract (W) aliquots, as described in Materials and Methods. The amount of *CEN6* DNA within IP samples was also measured using qPCR and the values compared to estimates for the equivalent locus derived from the calibrated ChIP-seq profiles (**D**) (see also Figure 5). Both sets of values are plotted on an arbitrary linear scale designed so that the ‘areas’ under each curve were identical. Note that *CEN6* occupancy rises and falls more rapidly than OR. Also shown in D are *CEN6* values calculated from uncalibrated data.

To show that our calibration method can be used in a realistic experimental setting, we used it to measure changes in cohesin's association with *S. cerevisiae* genomes as cells transit through the cell cycle. Cells arrested in early G1 by α-factor pheromone were triggered to enter S phase (Figure 3A) by removal of the pheromone and samples fixed every 15 min. A synchronous wave of budding took place 20–40 min after release and nuclear division occurred about 50 min later (Figure 3B); the latter being triggered by cohesin's cleavage by separase. Each fixed sample was then mixed with the same quantity of fixed cells from an exponential culture of *C. glabrata*. We chose this protocol in this particular case instead of mixing live cultures prior to fixation because of practical difficulties of maintaining two growing cultures while sampling from a synchronous culture. However, for reasons outlined in materials and methods this is not the optimum strategy and we have subsequently worked out a protocol to use live calibration cells even when taking samples from synchronous cultures.

Using data sets derived from whole cell extracts (W) and IPs (IP), we calculated the OR of *S.cerevisiae* cohesin for each time point. Consistent with previous estimates comparing cohesin's occupancy of defined loci using quantitative PCR to measure ChIP efficiencies, cohesin's OR was low in pheromone arrested cells, rose dramatically around the time or shortly before cells entered S phase, declined as cells underwent nuclear division, and rose again shortly before cells embarked on the next round of DNA replication (Figure 3C).

**Figure 4.**
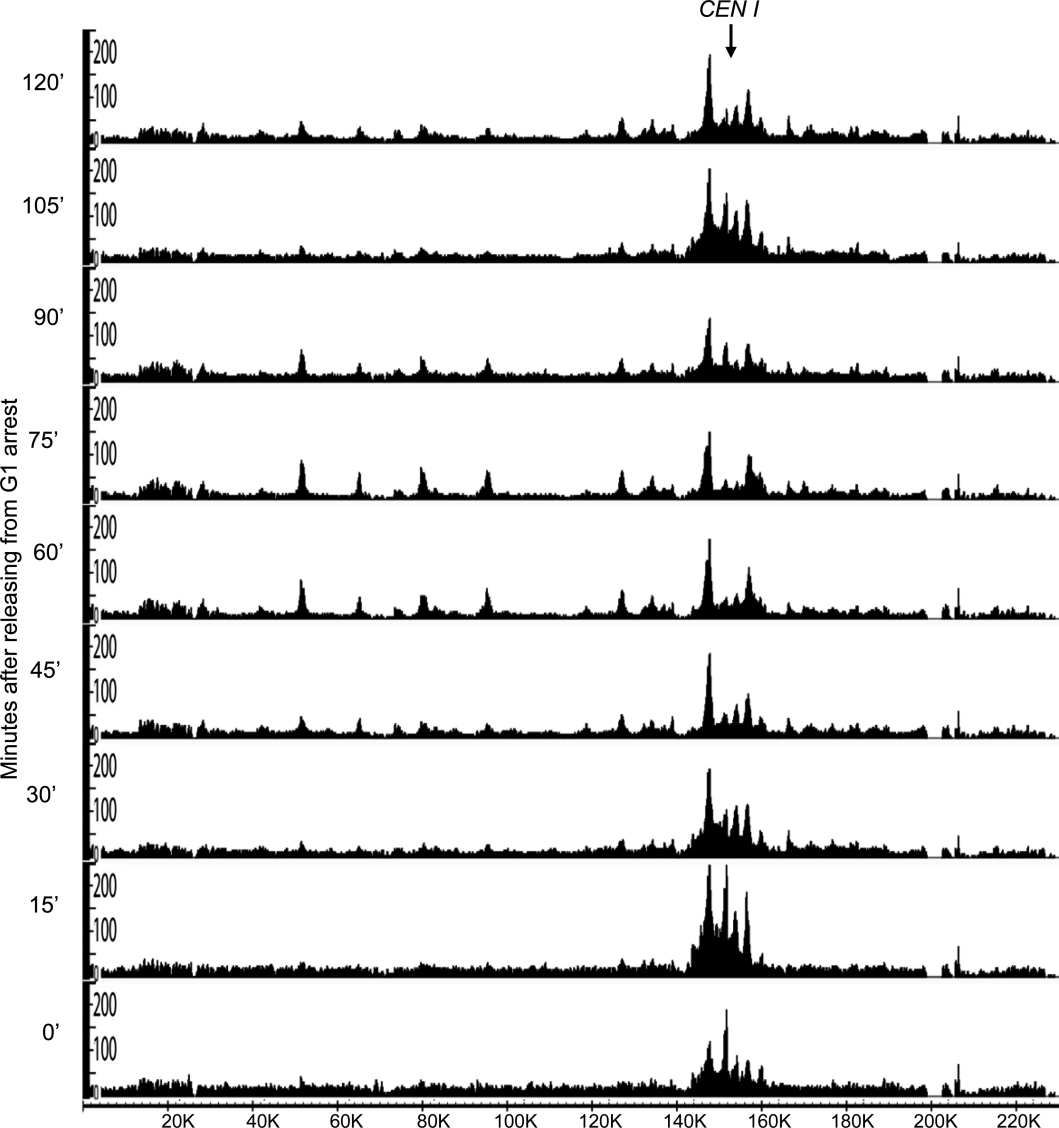
Conventional ChIP-seq profiles of cohesin (Scc1) during synchronous *S. cerevisiae* cell cycles. The ChIP-seq profile of Scc1 along chromosome 1 from each time point of the experiment described in Figure 3 shown on the basis of reads_per_million. Note that under these circumstances, which is the conventional way of presenting ChIP-seq data, the area under the curve (for the whole genome) will be identical between each sample. The Y-axis indicates the numbers of reads covering every base pair and the X-axis indicates position of every base pair adopting from SGD (http://www.yeastgenome.org).

**Figure 5.**
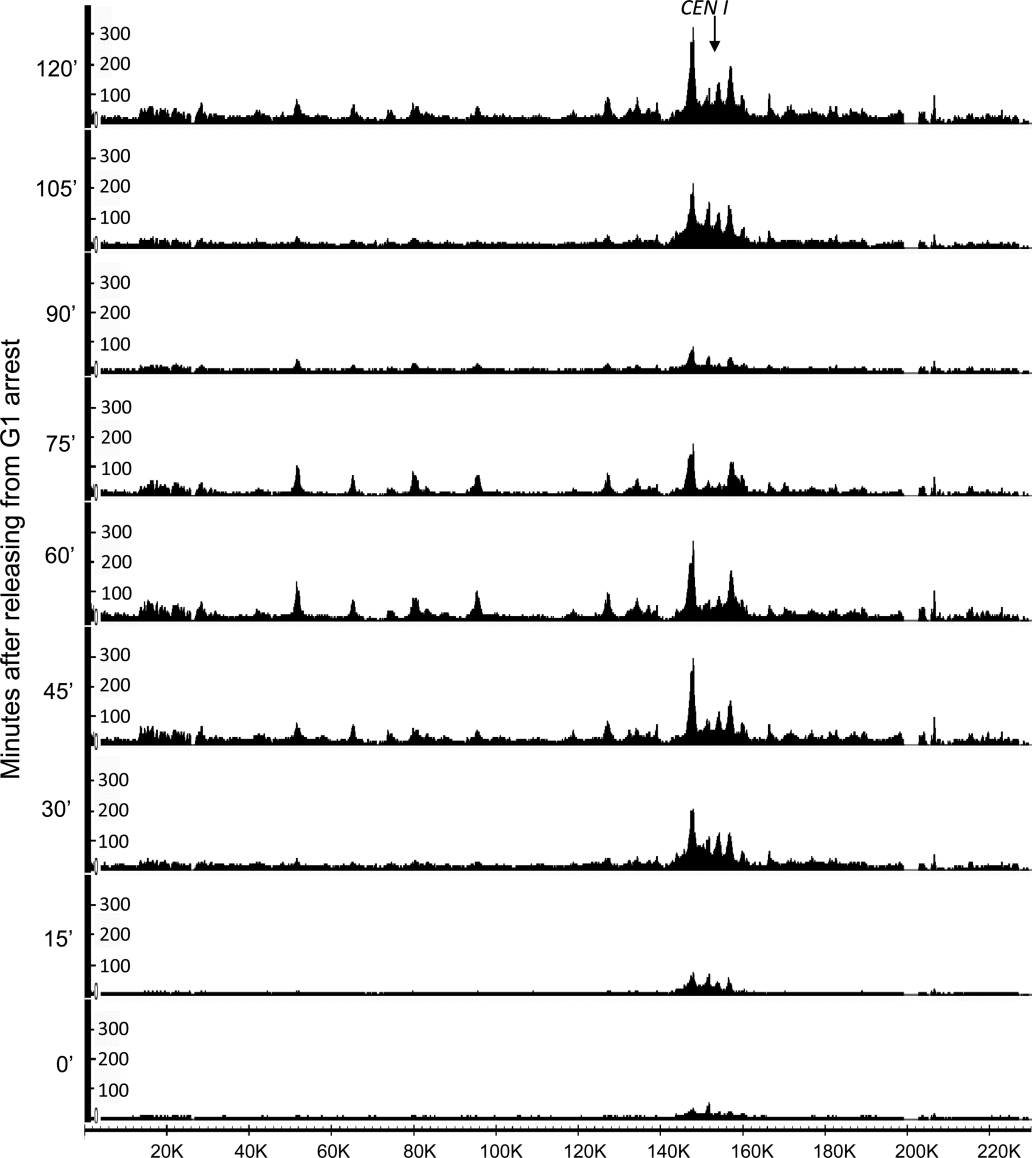
Cohesin chromodynamics: calibrated ChIP-seq profiles of Scc1 during synchronous *S.cerevisiae* cell cycles. The calibrated ChIP-seq profiles of Scc1 were calculated by multiplying the conventional ChIP-seq signals (Figure 4) with the OR values of each time point (Figure 3C). Unlike conventional ChIP-seq, the calibrated profiles reveal the changes in cohesin's occupancy of all sites within the genome as cells progress through their synchronous cell cycles. The Y-axis indicates the numbers of reads covering every base pair and the X-axis indicates position of every base pair adopting from SGD (http://www.yeastgenome.org).

Despite cohesin's low OR in pheromone arrested cells, shortly after release (15 min), and in cells that had just undergone nuclear division (90 min), uncalibrated ChIP-seq profiles revealed substantial association of cohesin with DNA sequences, especially in the neighbourhood of centromeres (Figure 4). Though never previously documented, some cohesin does associate with the genome, especially around centromeres in early G1 cells. However, because uncalibrated ChIP-seq profiles merely distribute reads along the genome, they are misleading as to cohesin's actual occupancy. Calibration of the ChIP-seq profiles of each time point using their ORs reveals that there is in fact rather little cohesin associated even with centromeres compared to post-replicative cells (Figure 5), that there is a huge increase by 30 min., when cells enter S phase, a large drop as cells undergo nuclear division, and re-accumulation shortly before cells re-enter S phase during the next cell cycle (Figure 5). The large increase in cohesin's association around centromeres shortly before cells enter S phase as documented by our calibrated ChIP-seq profiles is fully consistent with the accumulation around centromeres of GFP-tagged cohesin in living yeast cells shortly before bud formation (Supplementary Movie 1).

Hitherto, ChIP has been quantified using PCR to measure the amounts of DNA in IP samples compared to whole cell extracts (qPCR-ChIP), a method that is technically demanding and merely reveals information about occupancy at a single locus. To validate our calibrated ChIP-seq, we used qPCR to measure the amount of cohesin associated with sequences close to *CEN6* in aliquots from IP samples from each time point and compared changes in occupancy measured by this means with estimates of *CEN6* occupancy changes obtained from calibrated ChIP-seq. Crucially, the *CEN6* occupancy profiles were very similar but different from a *CEN6* occupancy profile calculated using ChIP-seq data that had not been calibrated (Figure 3D). This emphasizes the fallacy of trying to measure occupancy changes using uncalibrated ChIP-seq data.

Interestingly, the calibrated ChIP-seq and qPCR *CEN6* occupancy curves are both significantly different to the OR curve, which reflects occupancy throughout the genome. Cohesin accumulates at and disappears from *CEN6* more rapidly than for the genome in general. Close inspection of ChIP-seq profiles (whether calibrated or uncalibrated) reveals that cohesin associates with sequences around centromeres earlier than along arms, an effect that is especially prominent close to core centromeres and may be due to their key role in loading cohesin throughout a 50 kb peri-centric window (12,13). We return to this issue later in the paper. Also noteworthy is the observation that cohesin's accumulation within the intergenic regions between convergent genes along chromosome arms is not completed until cells finish S phase (60 min). This is significantly after association with chromatin in general (as measured by OR values) has reached maximal levels (45 min). If as suspected, the accumulation at such loci is due to translocation of cohesin from neighbouring sequences, its timing might be influenced by a reduction in cohesin's turnover on chromosomes brought about by acetylation of Smc3 during S phase (14,15).

Importantly, the calibrated ChIP-seq profiles from a second independent α factor release experiment revealed a similar pattern of genomic occupancy as cells underwent S phase (Supplementary Figure S6), demonstrating that the method is both reliable and robust. Our analysis of cohesin's genomic occupancy using calibrated ChIP-seq is the first example of measuring a protein's occupancy along a genome as cells transit between multiple cell states. Such ‘biological chromodynamic’ measurements will be a cornerstone of chromosome biology in the future.

Because of differential replication during S phase of early and late replicating sequences, more precise profiles in replicating cells would need to take into account DNA copy number across the genome. This could also have been achieved taking a second set of samples which had not been fixed with formaldehyde and thereby measuring the copy number of all genomic loci as the *S.cerevisiae* cells underwent DNA replication.

### Calibrated ChIP-seq and the background problem

**Figure 6.**
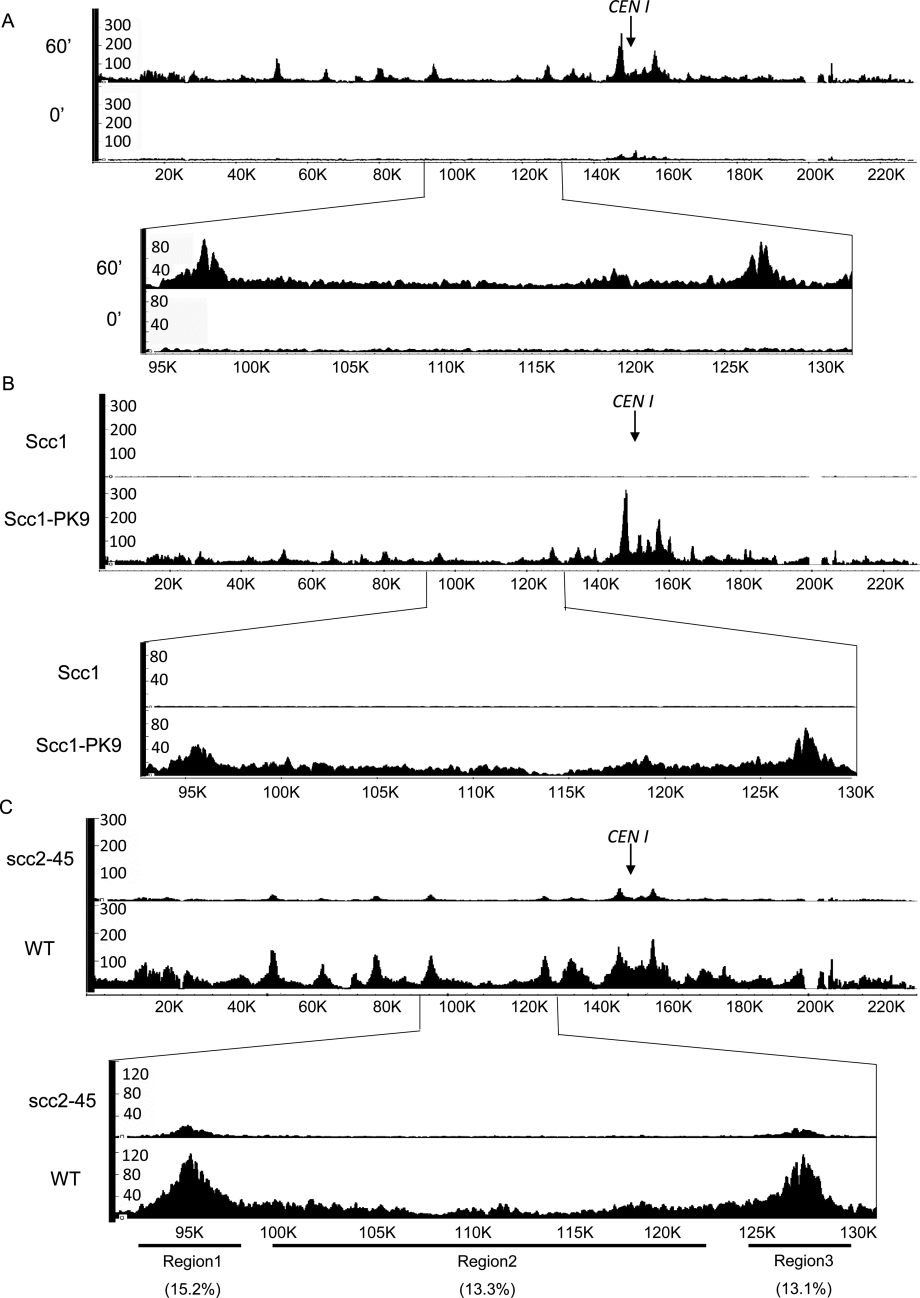
Calibrated ChIP-seq unlike conventional ChIP-seq distinguishes signals from noise. (**A**) Calibrated ChIP-seq profiles for chromosome 1 in pheromone arrested cells (0) and 60 min after release (60). (**B**) Calibrated ChIP-seq profiles for chromosome 1 obtained using the PK-specific antibody from exponentially growing *S. cerevisiae* cells with or without PK tagged Scc1 (K14601 or K699). To calibrate each sample, they were mixed with exponentially growing *C. glabrata* cells prior to fixation. (**C**) Calibrated ChIP-seq profiles of PK tagged Scc1 in the presence or absence of Scc2 activity. Wild type (K14601) and ts *scc2–45* cells (K22390) growing exponentially at 25°C were uniformly arrested in early G1 by incubation with α factor pheromone for 2.5 hour. The arrested *S.cerevisiae* cells were released into a new cell cycle by transferring to YPD media containing nocodazole at the restrictive temperature 37°C. After 60 min, by which time most cells had completed DNA replication, cells were fixed with formaldehyde and mixed with separately fixed *C. glabrata* cells before processing samples for ChIP-seq. The Y-axis indicates the numbers of reads covering every base pair and the X-axis indicates position of every base pair adopting from SGD (http://www.yeastgenome.org).

An important corollary of being able to measure quantitative changes in a protein's occupancy at all loci within a genome using epitope tagged proteins is that it enables one to evaluate whether association is real or merely background noise. This is particularly important for loci that are not within defined peaks. For example, the levels of cohesin associated with almost all chromosome arm sequences including those between peaks in cells that have entered S phase is clearly much higher than that observed in pheromone-arrested cells (Figure 6A), suggesting that these are real signals and not mere background (compare 0 and 60 min. samples in Figure 5 and Supplementary Figure S6D). To confirm this, we compared the calibrated ChIP-seq profiles of tagged and untagged cells. This revealed that the number of reads from the former was many times greater than the latter, whether or not they were associated with peaks or with intervals between peaks (i.e. troughs) (Figure 6B). This proves that the majority of reads throughout the genome are associated with the PK tag.

These data do not however exclude the possibility that reads within troughs are due to the adventitious association with chromatin of what is in fact soluble Scc1-PK within the nucleoplasm. If the latter were the case, then reads should be unaffected by mutations that inactivate the Scc2/4 complex necessary for loading cohesin onto chromosomes (16). To address this, we compared the calibrated ChIP-seq profiles of wild type *SCC2 SCC1-PK* and *scc2–45 SCC1-PK* cells following release at 37°C from a G1 arrest induced by growing cells in the presence of pheromone at 25°C. *scc2–45* is a temperature sensitive allele of *SCC2* that permits loading and cell proliferation at 25°C but not at 37°C. After incubation in pheromone free medium containing nocodazole at 37°C for 60 min, during which time both wild type and mutant cells completed DNA replication, samples were fixed with formaldehyde, mixed with fixed asynchronously growing *C. glabrata* cells, and subsequently processed for calibrated ChIP-seq. This revealed that the frequency of reads within troughs as well as peaks was greatly reduced by *scc2–45*, to between 13 and 15% of wild type (Figure 6C). Thus, the majority of reads within troughs as well as peaks arise from cohesin complexes whose association with the genome is dependent on Scc2.

Very similar results were obtained with the *scc2–4* mutation, with the only difference being that the latter reduced association to an even greater extent at 37°C (Supplementary Figure S7A). These results differ substantially from those obtained using microarrays (ChIP-chip) to measure cohesin's genomic distribution (Supplementary Figure S7B), which concluded that in *scc2–4* mutants cohesin associates with putative loading sites, which do not necessarily correspond to sites to which it subsequently relocates (17). The cohesin peaks in wild type cells observed in the ChIP-chip study resemble those observed in our calibrated ChIP-seq profiles. These peaks are largely absent in the ChIP-chip profiles from *scc2–4* cells but they are supposedly replaced by ones that coincide with peaks of Scc2 protein association (in wild type cells) (Supplementary Figure S7B). The point is that none of these new cohesin peaks specific to *scc2–4* mutants are observed in our calibrated ChIP-seq data (Supplementary Figure S7B). We suggest that the previous ChIP-chip study, which did not involve calibration and used inherently less sensitive microarrays instead of sequencing, may not have been able to distinguish background from signal.

In conclusion, our calibrated ChIP-seq reveals that the majority of reads, including those in troughs between peaks, are real signals. In other words, very real amounts of cohesin exist outside peaks. For all we know, this population may be as functionally significant as that within peaks, a feature that may apply to many other proteins whose functional properties has been inferred merely from the distribution of their peaks.

### Using calibrated ChIP-seq to analyse mutant proteins

**Figure 7.**
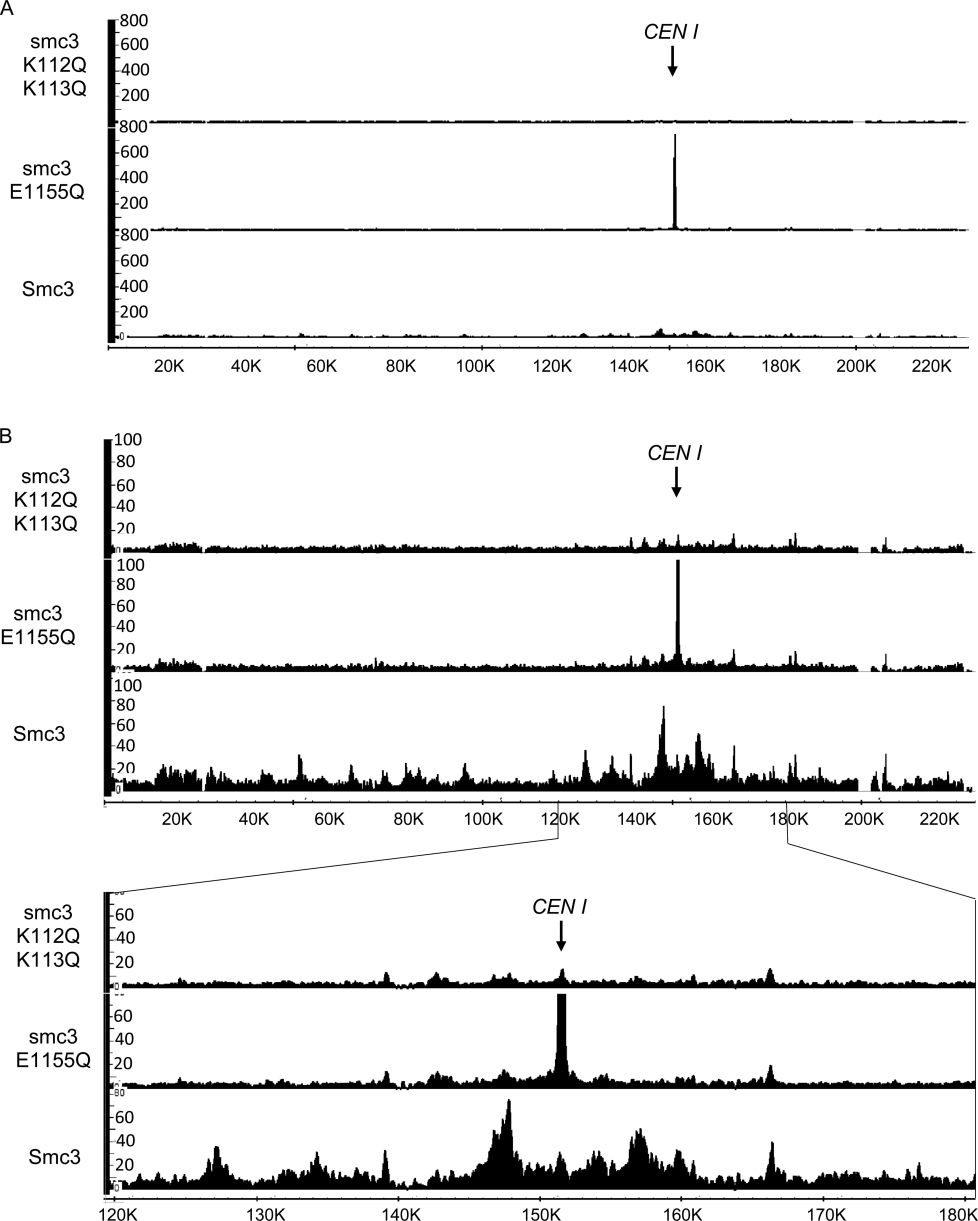
Calibrated ChIP-seq profiles of wild type, acetylation-mimicked mutant and ATPase hydrolysis mutant Smc3. The yeast cells ectopically expressing PK-tagged wild type Smc3 (K17407), acetylation-mimicked mutant smc3_K112Q, K113Q (K22703) or ATPase hydrolysis defective mutant smc3_E1155Q (K17409) were exponentially grown at 25°C and mixed with *C. glabrata* cells. Calibrated ChIP-seq was performed against PK epitope. (**A**) The calibrated ChIP-seq profiles of Chr 1 are shown with a full scale of Y-axis and the position of *CEN1* is indicated. (**B**) The enlarged DNA association profiles of WT and indicated mutant Smc3 are shown with a smaller scale of Y-axis. The detailed distribution of WT and mutant Smc3 at pericentromere (around 30 kb of flanking region on either side of *CEN1*) is shown underneath. The Y-axis indicates the numbers of reads covering every base pair and the X-axis indicates position of every base pair adopting from SGD (http://www.yeastgenome.org). Note that the E11155Q peak associated with *CEN1* has been truncated in (B).

An advantage of using tagged proteins to measure ChIP-seq profiles is that this makes possible the analysis of mutant proteins that are dysfunctional and cannot therefore sustain cell proliferation. One can compare the profiles of tagged wild type and mutant proteins when expressed from an ectopic locus in cells expressing untagged endogenous protein. To demonstrate this, we used calibrated ChIP-seq to measure the effect of mutations within another of cohesin's subunits, namely Smc3. Smc3E1155Q prevents hydrolysis of ATP bound to Smc3's ATPase domain. It greatly reduces cohesin's association with most parts of the genome but not association with core centromeres (13). Calibrated ChIP-seq revealed that PK tagged Smc3E1155Q was cross-linked to sequences in the immediate vicinity of core centromeres with a far greater efficiency than the corresponding wild type protein (Figure 7AB). This feature was missed by conventional ChIP-seq because the latter is not quantitative. It was also not picked up by ChIP quantitated by PCR (qPCR ChIP) because the latter used primer pairs slightly outside the Smc3E1155Q peak.

A second example is Smc3K112QK113Q, which replaces by glutamine a pair of lysine residues whose acetylation by Eco1 is essential for creating stable sister chromatid cohesion but not for cohesin's loading onto chromosomes (18–20). In wild type cells, Smc3K112 and K113 are thought to facilitate release of cohesin from chromosomes by a process mediated by its Wapl, Pds5 and Scc3 subunits. Acetylation of K112 and K113 by Eco1 is thought to block releasing activity and replacement of these residues by glutamine to mimic the effect of acetylation. If mutation of K112/3 merely inactivated Wapl-dependent releasing activity, then cohesin should still be functional and should load with reasonable efficiency onto chromosomes. Surprisingly, Smc3K112QK113Q is lethal and according to ChIP-qPCR reduces cohesin's association with a limited number of loci (21). Calibrated ChIP-seq revealed that the double mutation greatly reduced Smc3's association throughout the genome but more so in peaks than between peaks (Figure 7AB). One interpretation of this finding is that as well as inactivating cohesin's releasing activity, acetylation of K112 and K113 inhibits a productive interaction between cohesin and its Scc2/4 loading complex and K112QK113Q mimics this effect. However, in contrast with wild type where cohesin previously associated with chromatin is acetylated and thereby stabilized on chromosomes, K112QK113Q would affect the behaviour of the soluble pool and thereby compromise its ability to load in the first place. In conclusion, calibrated ChIP-seq can shed important insights into the behaviour of mutant proteins, insights that are not possible merely from inspection of conventional ChIP-seq profiles.

### A novel insight into the origin of peri-centric cohesin revealed by calibrated ChIP-seq

The surest way of showing that a new method is robust, reliable, and superior to existing ones is to make discoveries that were not previously possible. It is currently supposed that loading of cohesin onto chromosomes is mediated by its entrapment of chromatin fibres and given the large size of cohesin's tripartite rings, it has always seemed plausible that they could translocate (diffuse) along fibres subsequent to entrapment. Despite a number of studies, it has never actually been proven that this actually happens (17,22). The clearest example of a phenomenon that could be explained by such an activity concerns the origin of peri-centric cohesin in yeast. Accumulation of especially high levels of cohesin in a 50 kb window surrounding core centromeres depends on specific non-essential subunits of the kinetochore COMA complex and on core centromeres (23,24). Remarkably, transfer of a 120 bp core centromere from its endogenous site to an ectopic one on a chromosome arm not only eliminates high levels of cohesin around the deleted locus but also elevates cohesin levels throughout a 50 kb window surrounding the ectoptic site (12,13,25). One explanation for this phenomenon is that the COMA complex situated at core centromeres greatly facilitates Scc2/4-mediated loading of cohesin at this location and that once loaded these rings translocate into neighbouring peri-centric loci. An alternative is that COMA somehow modifies in a hitherto mysterious manner the chromatin structure for 20 kb on either side of core centromeres in a manner that facilitates loading throughout the modified region. Two pieces of evidence favour the first hypothesis (13). First, live cell imaging as well as conventional ChIP-seq shows that Scc2/4 accumulates, albeit with a very rapid turnover, at core centromeres. Second, cohesin containing Smc subunits that can bind but not hydrolyze ATP (e.g. Smc3E1155Q) accumulates (also fleetingly) at core centromeres, as if it undergoes an early step in a loading process that takes place at core centromeres but cannot complete the process, cannot entrap chromatin fibres, and cannot therefore translocate along them away from the initial site of loading.

**Figure 8.**
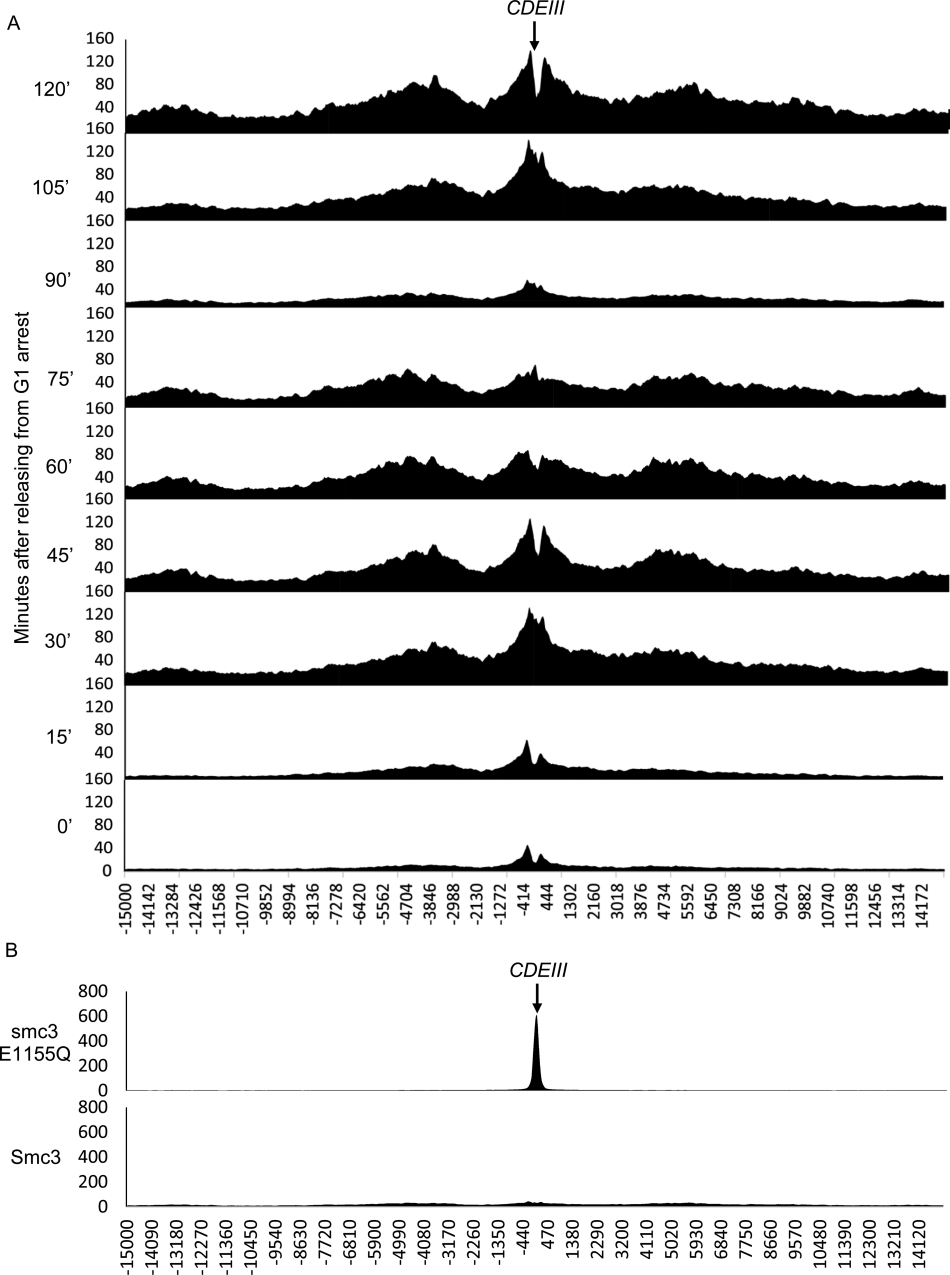
Cohesin dynamics around the centromere. The number of reads per base pair (Y-axis) in the 15kb regions either side of the *CDEIII* element for each chromosome (X-axis) were averaged and calibrated to reveal the average cohesin distribution around the centromere. (**A**) The profiles of the average cohesin distribution throughout the cell cycle from the data in Figure 3. (**B**) The average cohesin distribution in cycling Smc3 E1155Q ATP hydrolysis mutant cells compared to WT cells.

Crucially lacking, however, is any direct evidence that the cohesin which accumulates within peri-centric chromatin had actually loaded at core centromeres. In other words, no study has yet documented especially high levels of cohesin at core centromeres shortly after loading. Live cell imaging (Supplementary movie1) is consistent with this but has insufficient resolution to be certain. To address this issue, we analysed in greater detail cohesin's distribution around centromeres as cells passage through the cell cycle. To do this, we calculated at different stages of the cell cycle described in Figure 3 the average occupancy of cohesin at all positions 15 kb either side of *S. cerevisiae's* 16 core centromeres, which were aligned with base pair accuracy according to their highly conserved CDEIII elements. Figure 8A shows that in pheromone arrested cells, a large fraction of cohesin within this 30 kb interval is concentrated in two peaks immediately surrounding core centromeres. It is worth noting that this population of cohesin is only transiently associated with chromatin partly because of continual Wapl-dependent release (14) and partly due to separase cleavage activity that lingers in pheromone arrested cells (11). Occupancy is low presumably because Scc1 synthesis is also low but not negligible at this stage of the cell cycle. The transient nature of cohesin's association in these cells raises the possibility that cohesin resides on chromosomes for insufficient time to translocate away from its loading sites, raising the chances that it is detected at loading sites. The finding that a high fraction of peri-centric cohesin is actually detected in a bimodal peak on either side of the core centromere is therefore a further indication that loading dependent on core centromeres actually takes place at this site and not in neighbouring sequences.

The changes in cohesin's peri-centric occupancy when the arrested cells re-enter the cycle upon transfer to pheromone free medium is equally if not more revealing. Cohesin's occupancy in close proximity to core centromeres increases dramatically during the first 30 min after release, at the end of which period most cells have just entered S phase following a burst of Scc1 synthesis in late G1. Because of this increase, we can say with certainty that most of this cohesin must have loaded recently on the chromatin. Thus, a high fraction of cohesin that has just been loaded within a 30 kb peri-centric interval is found in very close proximity to core centromeres themselves. Thereafter, cohesin gradually accumulates in two broad peaks about 5 kb away from core centromeres and the amount associated with the latter actually declines. Separase activation, which triggers anaphase, removes cohesin from all loci between 75 and 90 min but this is followed by re-accumulation around core centromeres as a second round of Scc1 synthesis (which is accompanied by that of securin, which inactivates separase) takes place when cells enter a second cell cycle. The calibrated profiles portrayed in Figure 8A resemble a fountain that generates a wave of cohesin starting at core centromeres and moving into pericentric sequences. Importantly, no such changes were detected in profiles created from sequencing whole cell extracts (Supplementary Figure S8A).

When analysed in the same manner, cohesin containing Smc3E1155Q is found to concentrate precisely at CDEIII sites (Figure 8B), which is consistent with it engaging in an early step of the loading reaction but failing to entrap chromatin fibres and as a consequence dropping back off the fibre instead of translocating along it. Note that the distribution of wild type Smc3 in cycling cells (best seen in Supplementary Figure S8B) is as expected similar to that found in the G2/M phase cells from our synchronous culture (Figure 8A); most cells that accumulate high levels of cohesin in asynchronous *S.cerevisiae* cultures are in G2/M phase.

**Figure 9.**
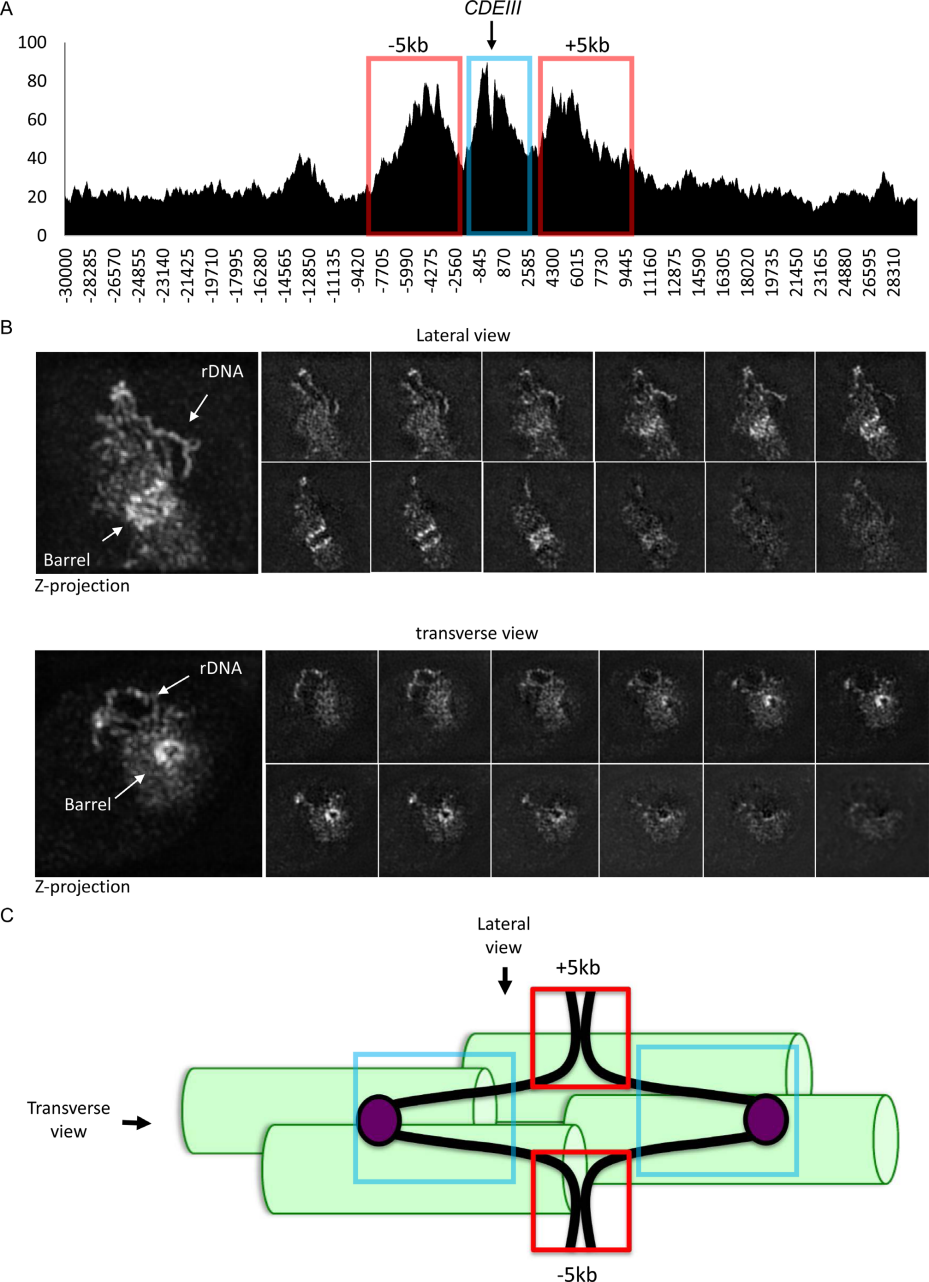
Pericentric cohesin viewed by calibrated ChIP-seq and cell images. (**A**) Cohesin's peri-centric distribution in G2/M phase cells. The number of reads per base pair (Y-axis) in the 30 kb regions either side of the CDEIII element for each chromosome (X-axis) were averaged and calibrated to reveal the average cohesin distribution around the centromere. The data was extracted from ChIP-seq of cells at 60 min. after released from G1-arrest in Figure 5. The ‘5 kb’ peaks are marked by red boxes and centromere-proximal cohesin marked by blue box. (**B**) High-resolution image of cohesin in G2/M phase. Tetraploid cells containing 64 chromosomes with endogenous Scc1 tagged with EGFP (K18719) were exponentially grown in YPD at 25°C and fixed with 2% of formaldehyde. The cells were observed under high-resolution OMX microscope. The lateral view is shown on the top panel and the transverse view on the bottom one. (**C**) Bi-orientation of sister kinetochores and peri-centric cohesion mediated by ‘5 kb’ peaks (red boxes) in G2/M phase cells. Sixty-four such bi-oriented chromosomes will be clustered around pole to pole microtubules, generating the cohesin barrels shown in (B). Chromosome axes – the black fibre. Kinetochores – mauve balls. Centromere-proximal cohesin - blue box. Pole to pole microtubules – green cylinders.

The accumulation of peri-centric cohesin in two broad peaks whose centres are 5 kb away from core centromeres is also observed when sequences 30 kb either side of all 16 core centromeres are aligned with each other (Figure 9A). The ‘5 kb’ peaks therefore represent a large fraction of the peri-centric cohesin recruited by COMA complexes and presumably therefore correspond to the ‘barrels’ of GFP tagged cohesin observed by high resolution microscopy in cells whose kinetochores have bi-oriented (see Figure 9B). Because loss of cohesin's peri-centric enrichment in COMA mutants is accompanied by reduced cohesion in the vicinity of centromeres (13,25), we presume that the peri-centric cohesin we observe in the ‘5 kb’ peaks (marked by red boxes in Figure 9A and C) is concerned with holding sister chromatids together. This contrasts with the situation for cohesin situated in the immediate vicinity of centromeres, which is associated with a part of the genome that is drawn towards opposite spindle poles when sister kinetochores bi-orient and will therefore be less dense and noticeable in cell images. We therefore suggest that the ‘barrels’ of GFP tagged cohesin seen in live cells following kinetochore bi-orientation arise due to the clustering of cohesin within peri-centric 5 kb peaks of all chromosomes around the central spindle (Figure 9C). This contrasts with a previous suggestion that the barrels are composed of cohesin associated with chromatin fibres that have been pulled apart from their sisters by the bi-orientation process (26).

Though our data do not formally prove that the cohesin which accumulates within the two broad peri-centric peaks (5 kb on either side of core centromeres) as cells enter G2 is composed of molecules that had loaded at core centromeres, the distributions nevertheless represent a striking affirmation of this concept and therefore represent by far the clearest evidence so far that cohesin translocates along chromatin fibres away from its sites of loading. Calibrated ChIP-seq has therefore revealed a process long suspected to exist but never previously documented. Evidence that cohesin really does translocate along chromatin fibres is of fundamental importance for the entire field of Smc/kleisin complexes because it raises the possibility that condensin has a similar property, which could explain how it generates the loops that make up mitotic chromosomes (27).

## DISCUSSION

We describe here a simple technique that makes it possible to calibrate ChIP-seq profiles in a manner that quantitates changes in the distribution of an epitope tagged protein throughout the yeast genome. Central to this method is the calculation of occupancy ratios (ORs) by mixing ‘experimental’ cells (in this case *S. cerevisiae*) with ‘calibration’ cells from a different species (in this case *C. glabrata*) that express a similarly tagged protein. In one of the cases described here, the proteins that were identically tagged in the two organisms were in fact orthologs and their immunoprecipitation properties should be very similar if not identical. Though desirable, as it reduces potential variation in cross-linking and IP efficiencies, the use of orthologs is not essential for the method to work. We demonstrate for instance that *C. glabrata* cells expressing PK tagged Scc1 protein can equally well be used to calibrate ChIP-seq profiles of another cohesin subunit, namely Smc3. In this case, the OR's of PK tagged Smc3 from *S.cerevisiae* were lower than that of PK tagged Scc1 (1.4 versus 0.56), partly because the former was expressed in the presence of untagged endogenous protein but possibly also because Smc3 might be less efficiently cross-linked to DNA than Scc1. However, this effect is systemic and does not therefore compromise the calibration process. We therefore envisage that our *C. glabrata* strain expressing PK-tagged Scc1 could also be used to calibrate ChIP-seq profiles of numerous PK-tagged chromosomal proteins in *S.cerevisiae*.

The notion of using internal reference genomes to quantitate ChIP-seq has been described in outline in the context of antibodies that recognize the same histone modifications or RNA polymerase epitopes in different species (4,5). Neither of these studies demonstrated that their method was truly quantitative. Moreover, both used antibodies against endogenous epitopes shared by the experimental and calibration genomes. However, this is only possible if the proteins/modifications in question are extremely conserved, which is rarely the case given that the DNA sequences of experimental and calibration genomes must differ sufficiently to assign most sequences to one or another genome. In contrast, the use of epitope-tagged proteins described here provides a method that can be used for any chromosomal protein in any organism. It may be worth pointing out that the same calibration principle lies behind quantitative mass spectrometry and could also be more widely used to calibrate RNA-seq data.

We demonstrate for the first time another crucial advantage of using epitope tagged proteins. By comparing tagged and untagged strains, it is possible to distinguish signal from noise throughout the genome, something that has never hitherto been achieved. This permitted us to show that much cohesin is associated with sequences between peaks as well as at them. Comparing the calibrated ChIP-seq profiles of tagged and untagged cells revealed that the background noise of cohesin profiles is very low. This might not be the case for other factors whose cross-linking to the genome is less efficient than cohesin. In such cases, the calibrated ChIP-seq profiles obtained with untagged strains could be used to subtract the background noise from profiles obtained with tagged strains.

Using CRISP-R technology, epitope tagging endogenous loci is now feasible for most types of cell as well as embryos. Moreover, by tagging both gene copies, it is feasible to assess the functionality of tagged proteins, as has long been the case in yeast. For these reasons, calibrated ChIP-seq using tagged proteins as implemented for the first time here is likely to be the future technique of choice. For purists who maintain that tagging a protein alters its properties, then it is always possible to use an antibody to a defined epitope within the natural protein and then use CRISP-R to modify the endogenous gene so that its protein is no longer recognized by the antibody (reverse tagging).

Our calibrated ChIP-seq method does not per se control for cases where the immunoprecipitation step fails for some reason. Under such circumstances, OR values would obviously not be revealing about occupancy. We suggest two solutions to this problem. The simplest is to include a tagged versus untagged data set along with all other experimental samples when using new immunoprecipitation reagents. A more complex solution would be to include a third untagged genome into the calibration process, whose abundance in IP samples would reveal the signal to noise ratio.

We note that our method could also be used to quantitate modifications of chromosomal proteins even when antibodies specific for those modifications are species-specific. For example, humanization of the sequences around K113 in *S. cerevisiae* has little or no effect on Smc3 function but enables antibodies raised against modified human peptides to recognize acetylated K113 in *S. cerevisiae* (data not show). Humanizing *C. glabrata* in the same manner would allow one to calibrate the ChIP-seq profiles of acetylated Smc3 in *S. cerevisiae* cells (whose K113 region had been humanized) using the human-specific antibody.

There are an infinite number of experimental/calibration combinations that can be used. It is merely important that the experimental and calibration organisms are sufficiently similar that the precise ChIP protocol works for both. It is also possible to invert the roles of ‘experimental’ and ‘calibration’ organisms. For example, it is not inconceivable that *S. pombe* cells could be used to calibrate *S. cerevisiae* experiments and vice versa. Thus, these two heavily used model microorganisms could be used to calibrate experiments from each other. Calibrated ChIP-seq as described here largely obviates any need to measure occupancy using conventional quantitative ChIP using qPCR, a technique that merely reveals individual loci and is costly, time consuming, and technically demanding. Calibrated ChIP-seq will therefore have wide-ranging uses for chromosome biologists. We suggest that biological chromodynamics would an appropriate term for the measurement, using techniques like calibrated ChIP-seq, of changes in the occupancy of proteins (or their modifications) throughout genomes, be they in response to changes in the environmental, physiological, developmental, pharmacological, or genetic state of cells.

## Supplementary Material

SUPPLEMENTARY DATA
