# Supplementary material for: Biological chromodynamics: a general method for measuring protein occupancy across the genome by calibrating ChIP-seq
Source: Nucleic Acids Res. 2015 Jun 30;43(20):e132. doi: 10.1093/nar/gkv670 (PMC4787748; doi:10.1093/nar/gkv670)
Supplement: SUPPLEMENTARY DATA [file supp_43_20_e132__index.html]

Biological chromodynamics: a general method for measuring protein occupancy across the genome by calibrating ChIP-seq — SUPPLEMENTARY DATA 

# Biological chromodynamics: a general method for measuring protein occupancy across the genome by calibrating ChIP-seq

## SUPPLEMENTARY DATA

- SUPPLEMENTARY DATA
- SUPPLEMENTARY DATA
