# Supplementary material for: Biological chromodynamics: a general method for measuring protein occupancy across the genome by calibrating ChIP-seq
Source: Nucleic Acids Res. 2015 Jun 30;43(20):e132. doi: 10.1093/nar/gkv670 (PMC4787748; doi:10.1093/nar/gkv670)
Supplement: SUPPLEMENTARY DATA [file supp_gkv670_nar-00886-met-k-2015-File003.pdf]

Supplementary Figures 1

A

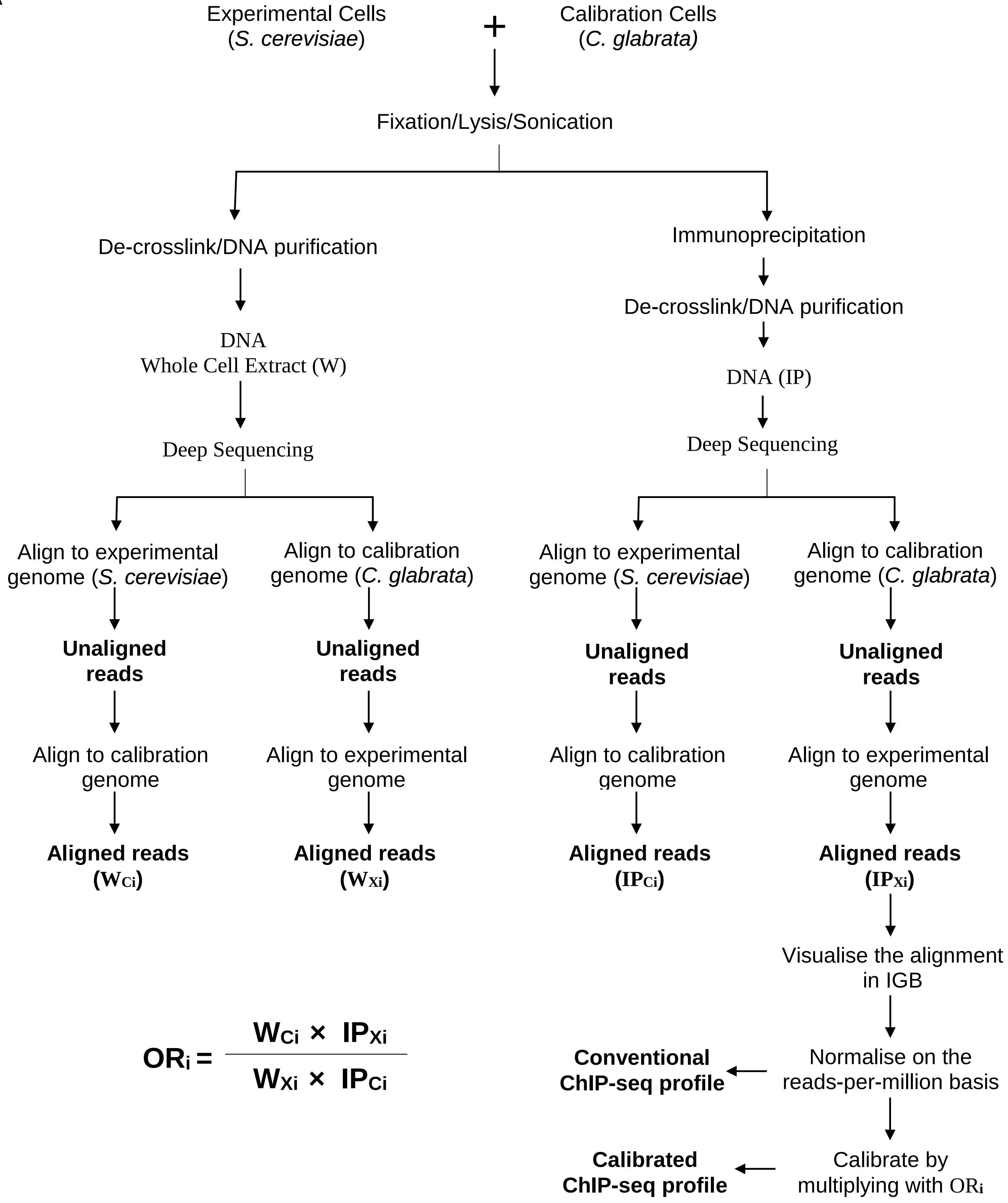

B

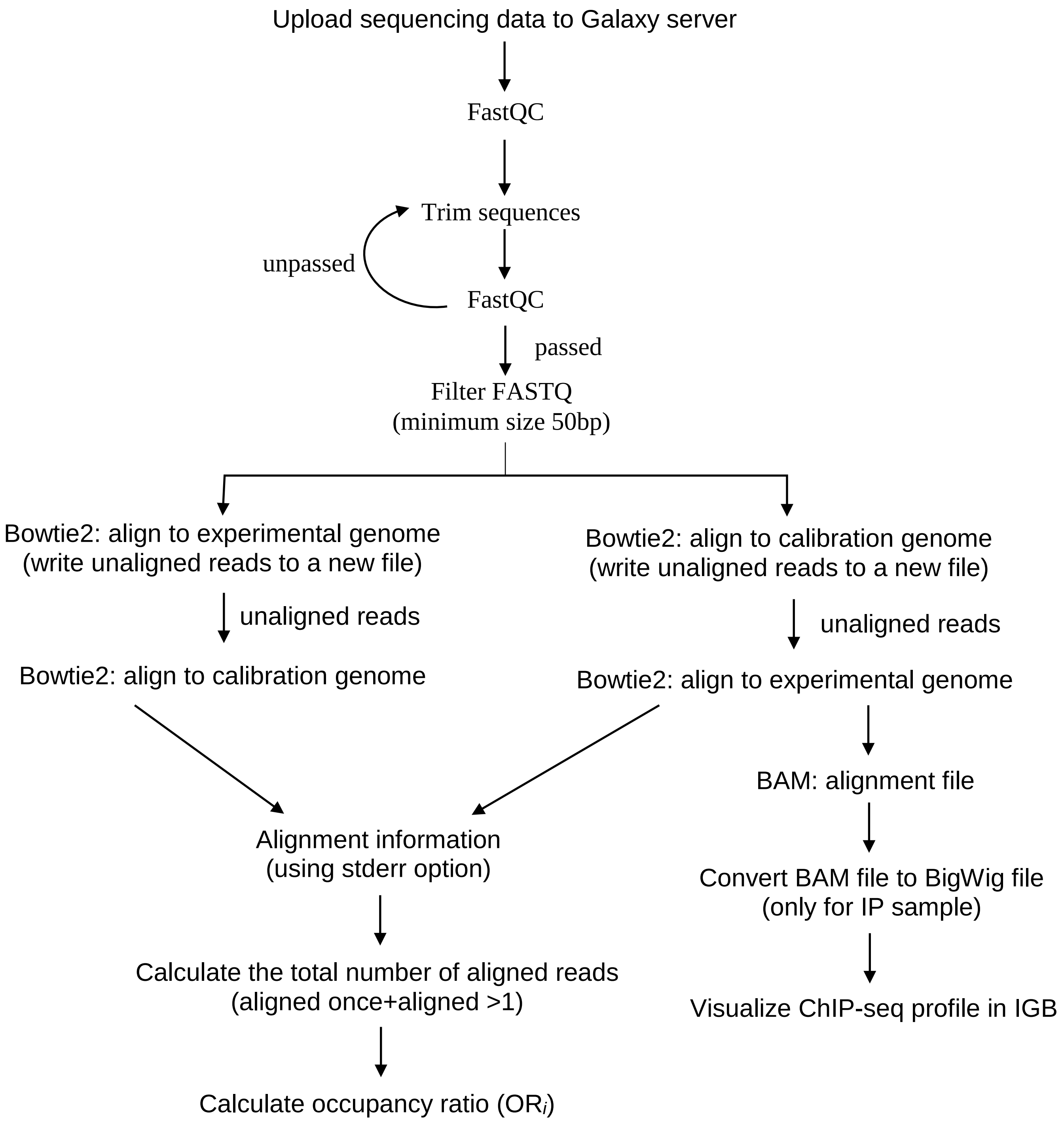

Supplementary Figures 1. (A) Flow scheme of the calibrated ChIP-seq procedure. (B) Analysis pipeline of sequencing data using Galaxy platform

Supplementary Figures 2

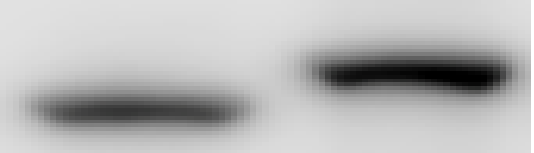

Supplementary Fig 2 Expression of Scc1PK9 in *Saccharomyces cerevisiae* and *Candida glabrata* cells. Proteins were extracted from exponentially grown yeast cells and resolved by SDS-PAGE. The Scc1PK9 were detected using anti-PK antibody.

Supplementary Fig 3

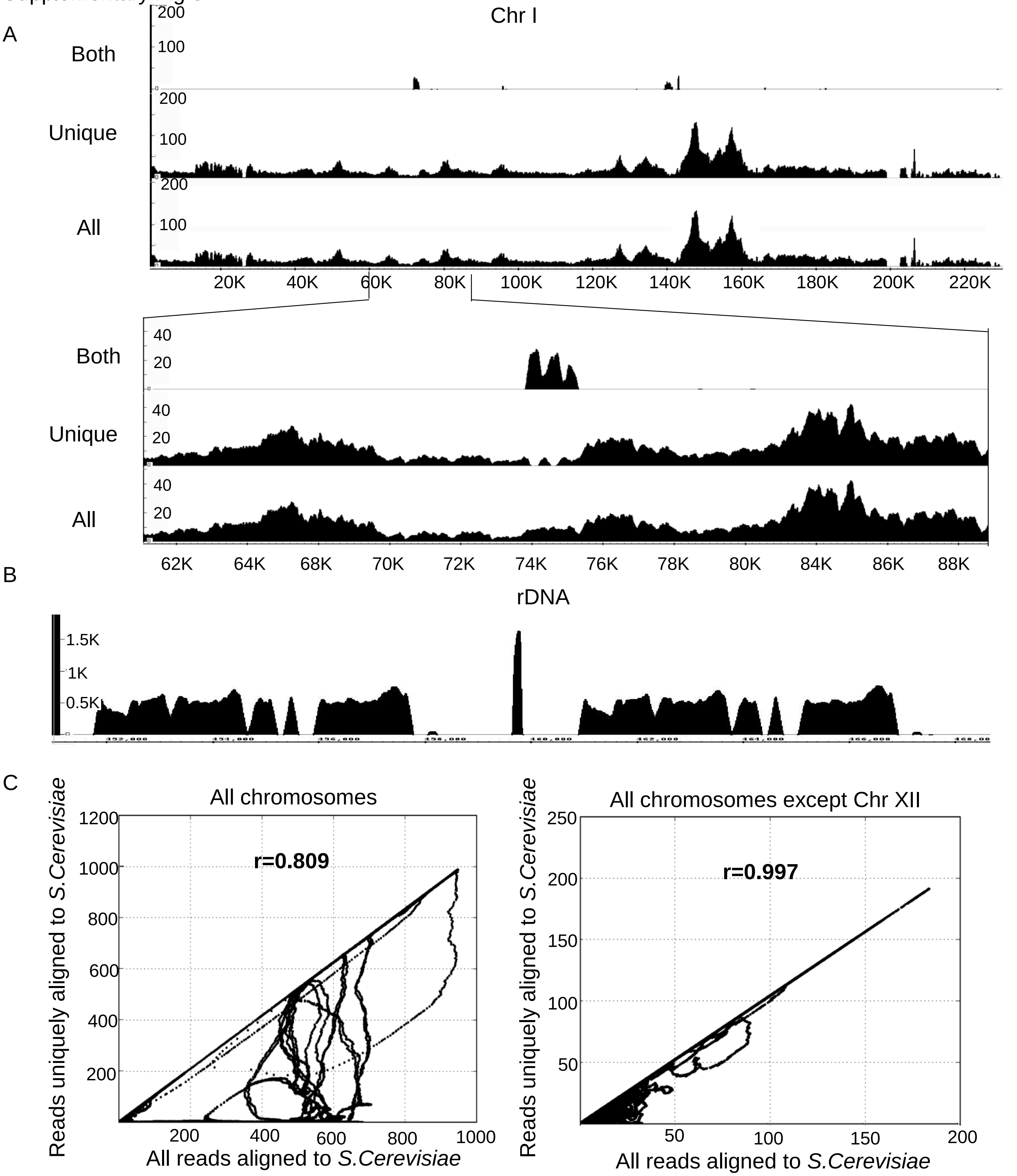

Supplementary Fig 3.

Regions within the *S.cerevisiae* genome whose sequences align with those of *C.glabrata*. (A) ChIP-seq sequences from *S.cerevisiae* only that align to both *S.cerevisiae* and *C.glabrata* genomes mapped onto chromosome 1 from *S.cerevisiae* (top), ChIP-seq sequences from *S.cerevisiae* only that align only to *S. cerevisiae* (middle), and ChIP-seq sequences from *S. cerevisiae* only mapped onto its chromosome I (bottom). All on the basis of reads per million. The overall profile of Chr I shown on the top three panels and a more detailed profile of a region (62,000-88,000) shown in the bottom panels. (B) ChIP-seq sequences from *S.cerevisiae* only that align to both genomes mapped onto rDNA region on chromosome XII from *S.cerevisiae*. (C) Correlations between ChIP-seq signals derived from all reads and those that

align only to *S. cerevisiae*. The numbers of reads assigned to each base pair of all the chromosomes (left) or all the chromosomes except Chr XII (right) in the alignment using sequences only aligned to *S. cerevisiae* were plotted against those using all the sequences.

Supplementary Fig 4

A

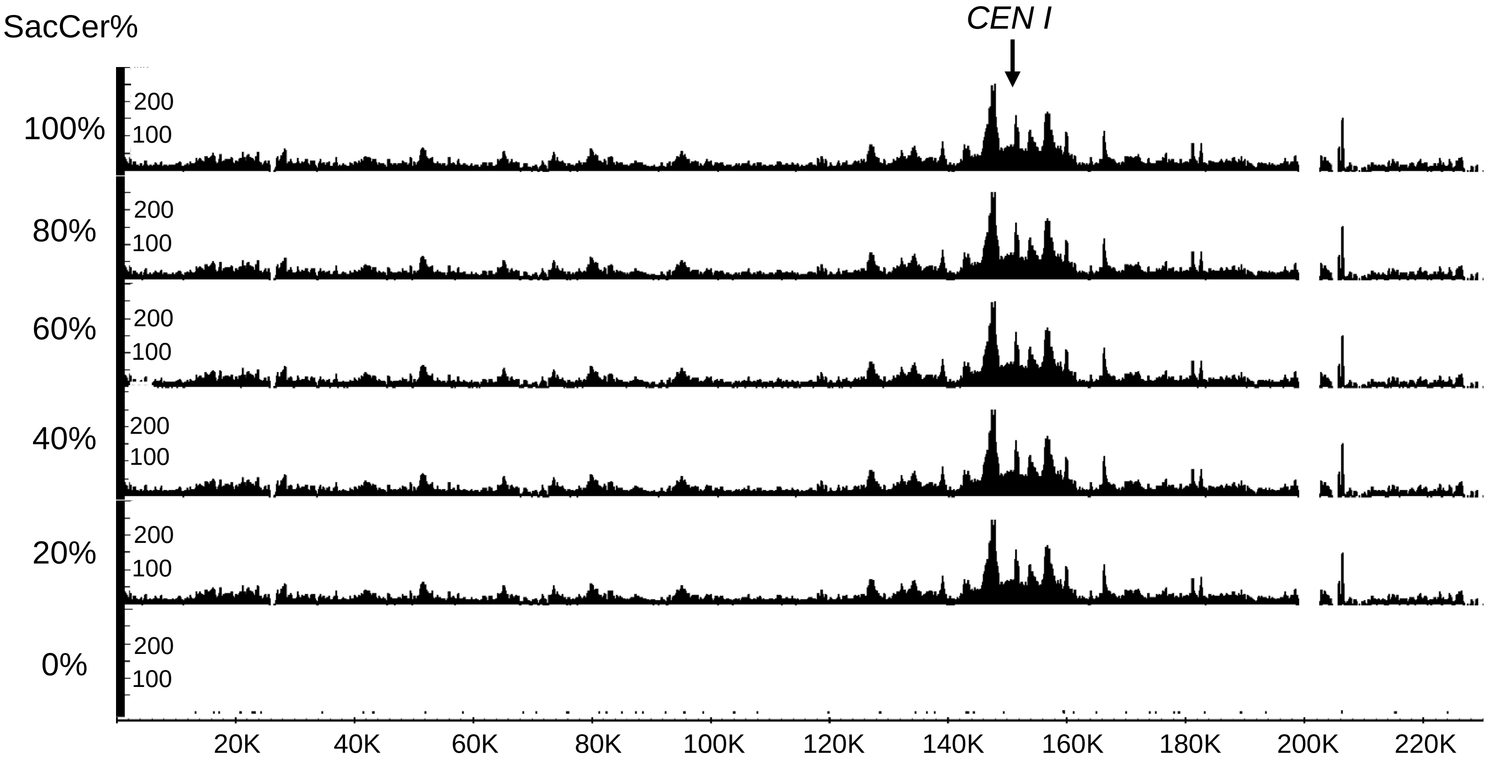

B

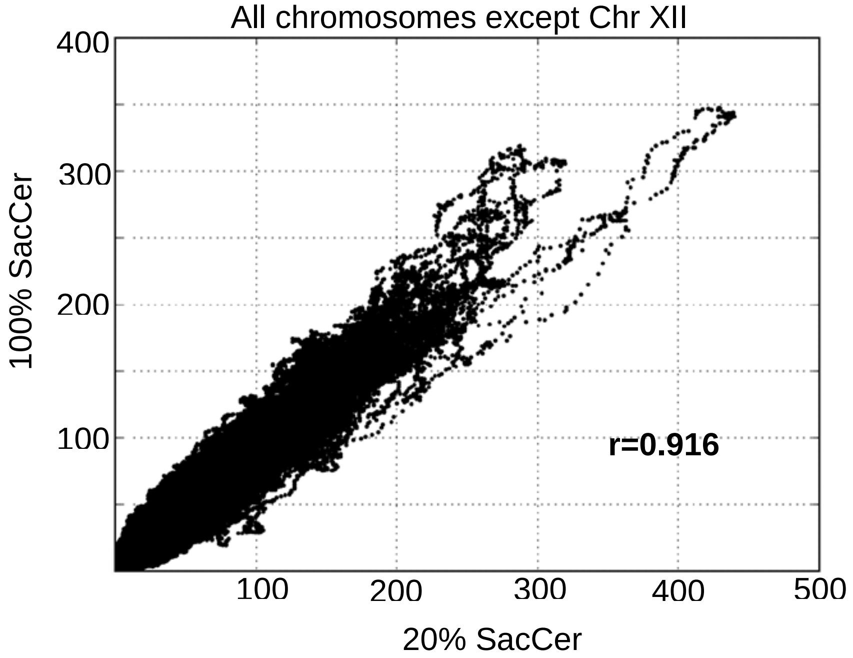

Supplementary Fig 4.

(A) Calibrated ChIP-seq profiles obtained by multiplying the uncalibrated profiles from Fig. 3C by the corresponding ORs. (B) Correlations between ChIP-seq signals derived from SacCer pure culture (100%) and from 20% SacCer mixture. ChIP-seq signals of each base pair except Chr XII bearing rDNA obtained from SacCer pure culture (100%) were plotted against those from 20% SacCer mixture.

Supplementary Fig 5

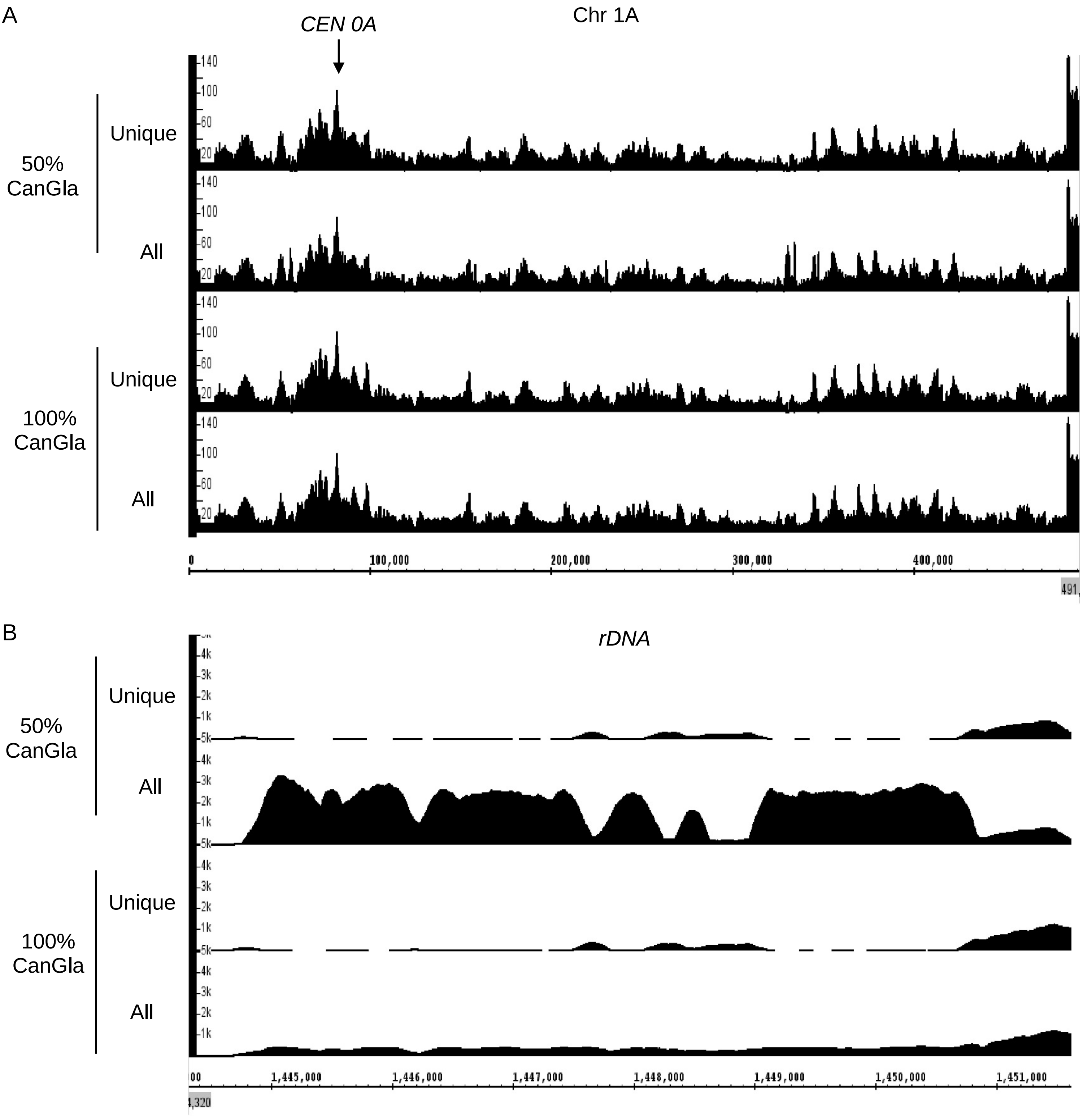

Supplementary Fig 5.

Sequences obtained from ChIP-seq shown in Fig1 B aligned to the *C.glabrata* genome. The distribution of CanGla_Scc1 on chromosome 1A (A) or rDNA region (B), using alignment either with all reads from a pure *C.glabrata* ChIP-seq or reads unique to *C.glabrata* from ChIP-seq from a *C.glabrata/S.cerevisiae* mixture.

Supplementary Fig 6

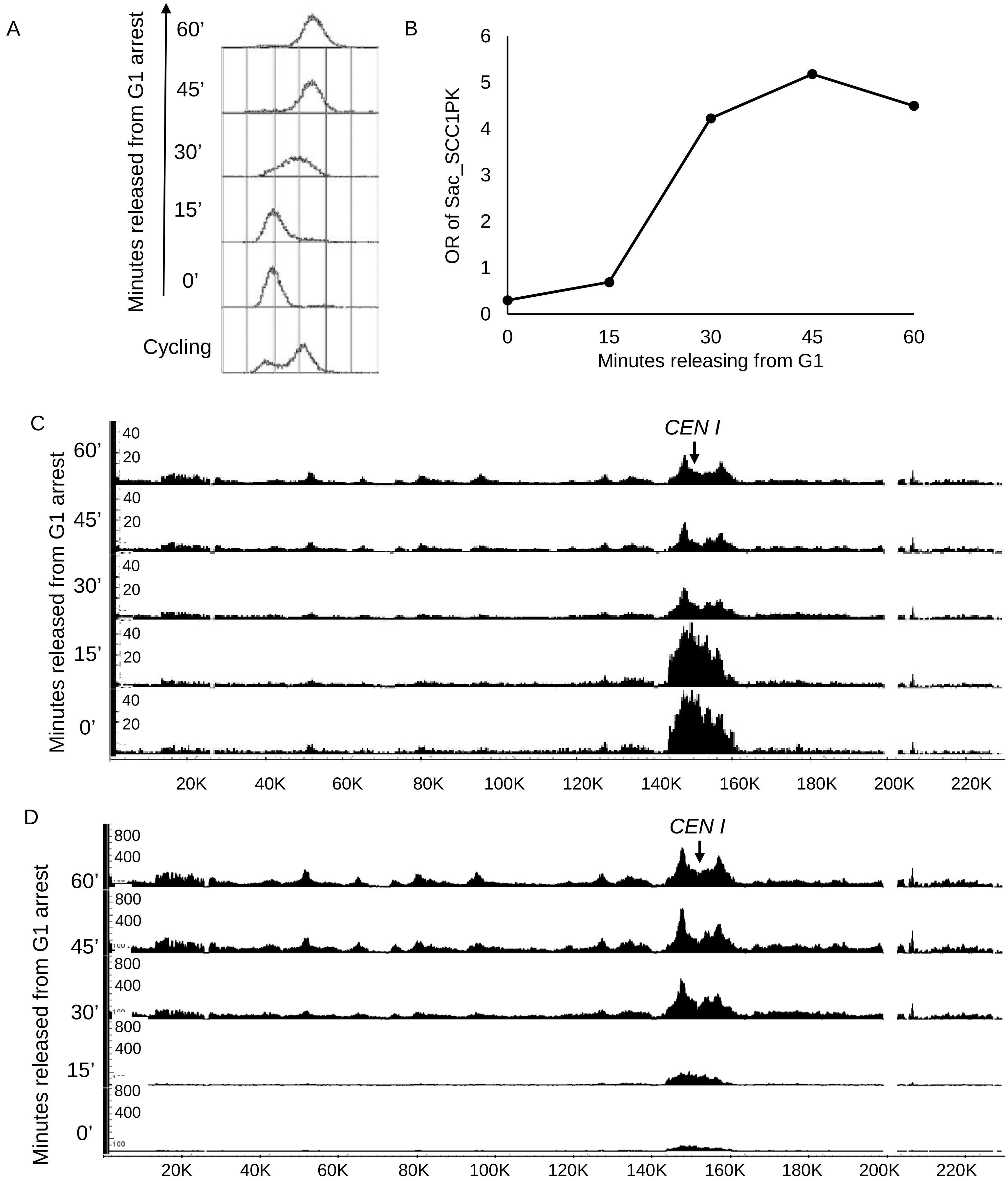

Supplementary Fig. 6.

Exponential phase *S.cerevisiae* cells (K14061) were arrested in G1 with α-factor and allowed to re-enter the cell cycle by transferring cells to pheromone free YPD media containing nocodazole, which prevents nuclear division. Samples of fixed cells with mixed with pre-fixed *C.glabrata* cells (K23308) and processed for ChIP-seq. (A) DNA content shown by FACS. (B) ORs from each time point after release. (C) Uncalibrated ChIP-seq profile of Scc1 on chromosome 1. (D) Calibrated ChIP-seq profile of Scc1 on chromosome 1.

Supplementary Fig 7

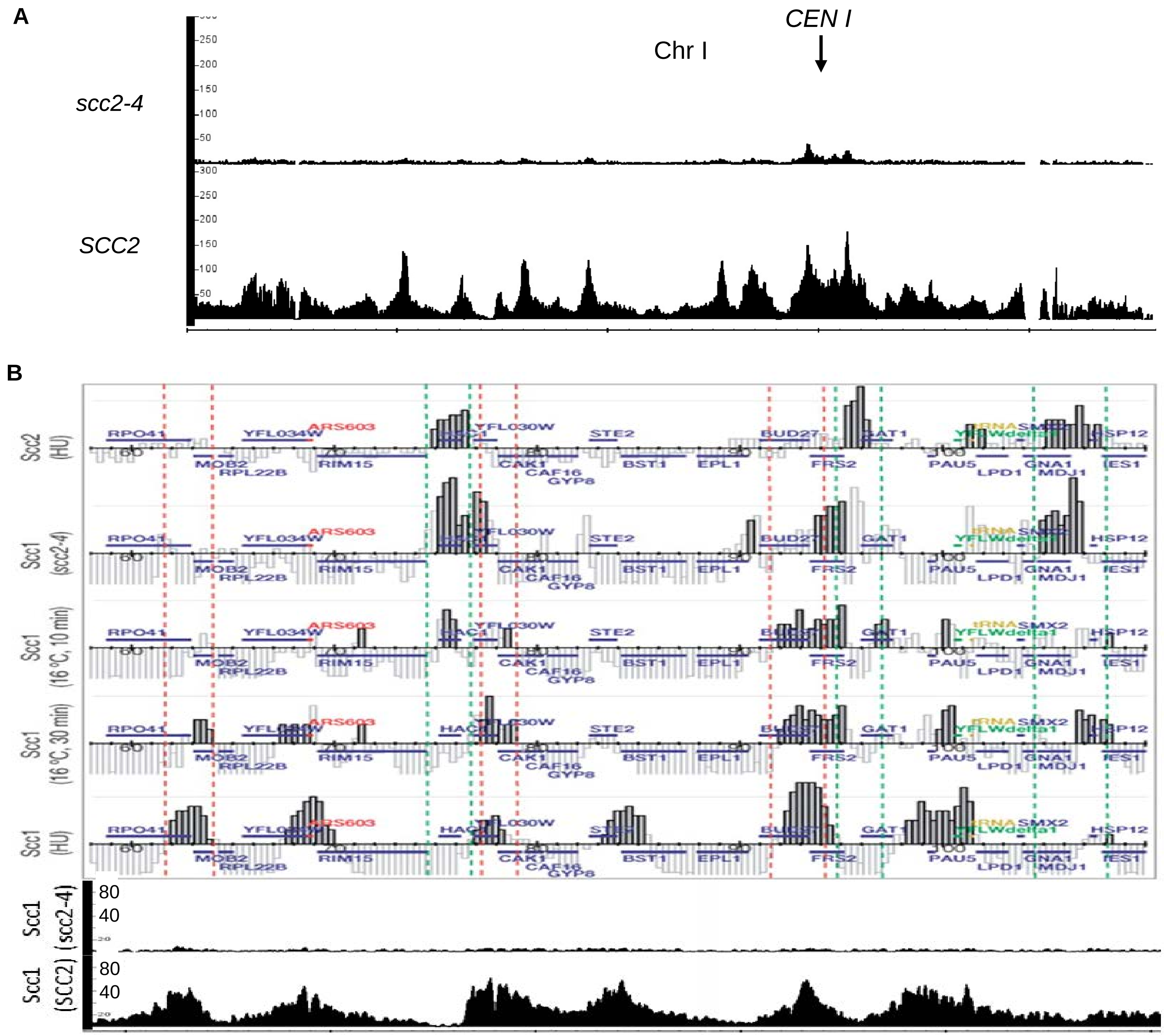

Supplementary Fig 7 Calibrated ChIP-seq profile of Scc1 in the presence or absence of Scc2. G1-arrested *S.cerevisiae* cells with wild type *SCC2* (K14601) or a ts allele *scc2-4* (K14710) were released into YPD media containing nocodazole at the restrictive temperature. After 60 minutes, cells were fixed, mixed with fixed *C.glabrata* cells and calibrated ChIP-seq performed. (A) Calibrated ChIP-seq profiles of chromosome 1. (B) A comparison of the ChIP-microarray data from Lengronne *et al.*, 2004 with the calibrated ChIP-seq data. The first five panels were adapted from Fig 3C in Lengronne *et al.*, 2004. Detail of the localisation on the left arm of chromosome VI, from top to bottom, of Scc2-HA6 in hydroxyurea-arrested cells, Scc1-HA6 in *scc2-4* mutant cells released from G1 into hydroxyurea block at restrictive temperature, Scc1-HA6 in wild-type cells 10 min, and 30 min following release from G1 at 16°C, and Scc1-HA6 in hydroxyurea-arrested wild-type cells. Enrichment in the immunoprecipitated fraction relative to a whole genome DNA sample is shown along the length of the chromosome. Each bar represents the average of 16 oligonucleotide probes within adjacent 300 bp windows. The y-axis scale is log2. Dark grey signals represent significant binding. The green and red dashed lines flank sites usually seen bound by Scc2 and Scc1, respectively. The last two panels are the corresponding calibrated ChIP-seq profiles of Scc1 in wild type and *scc2-4* cells strains described in A. Gratifyingly, the peaks observed by us in wild type cells using calibrated ChIP-seq (bottom panel) correspond to those observed by Lengronne *et al*. Note however that our scale is linear and theirs log2. According to Lengronne *et al*., these peaks largely disappear in *scc2-4* mutants but are replaced by others that co-incide with peaks of Scc2 binding in wild type cells. This phenomenon is not observed in our calibrated ChIP-seq profiles, implying that they are artefacts caused by background noise, which could not be measured using the uncalibrated technique used by Lengronne *et al*.

Supplementary Fig 8

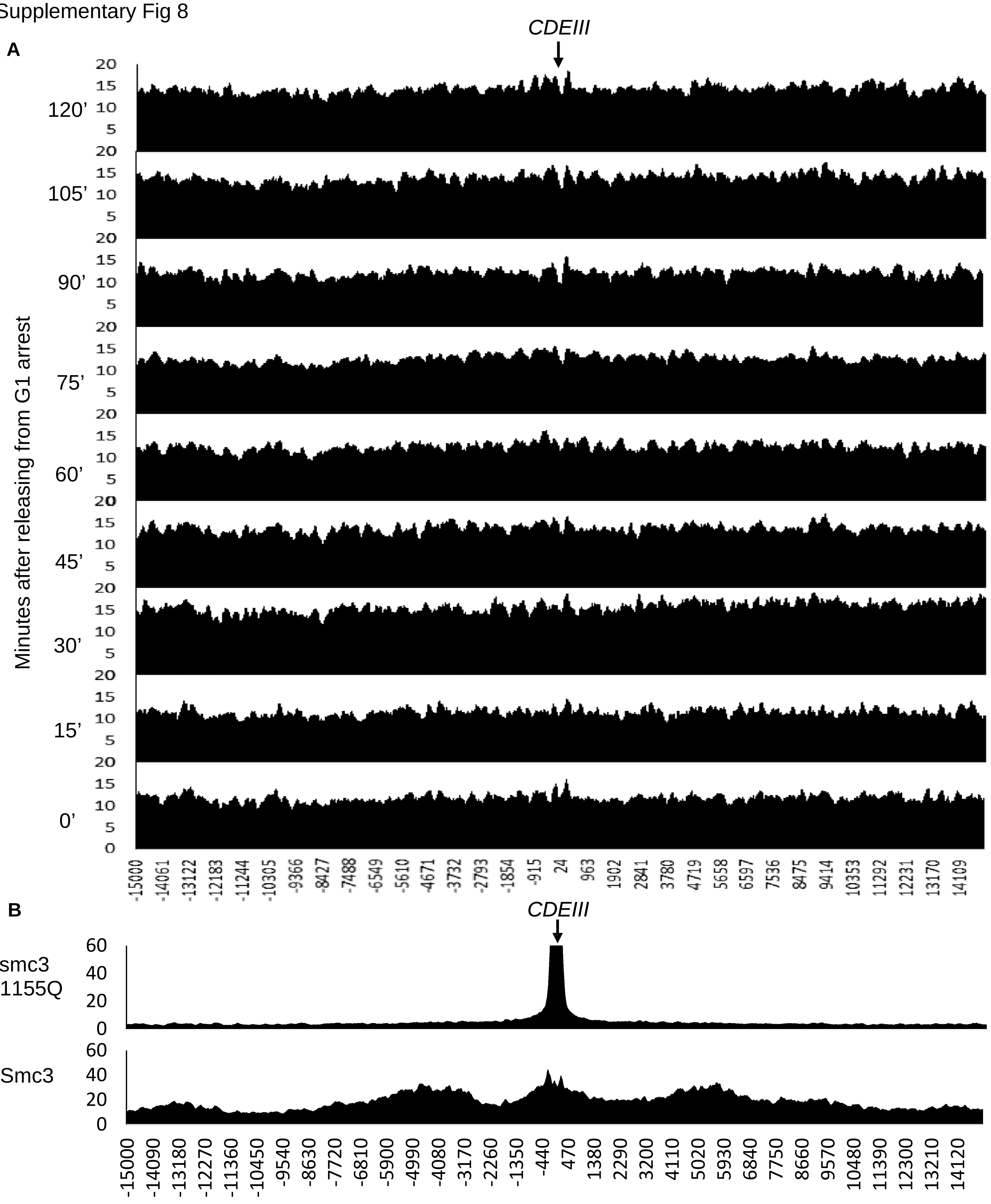

Supplementary Fig. 8.

(A) DNAs from whole cell extracts (W) in the experiment described in Fig 3 were sequenced and aligned to *S.cerevisiae* genome. The number of reads per base pair (Y-axis) in the 15kb regions either side of the CDEIII for each chromosome (X-axis) were averaged. (B) The detailed cohesin distribution in cycling Smc3 WT and E1155Q ATP hydrolysis mutant cells with a smaller Y-axis scale from Fig 8B. The E1155Q peak has been truncated.

Suppelmentary movie 1

Tetraploid yeast cells in which endogenous Scc1 or Mtw1 was fused with EGFP and mCherry respectively (K18719) were grown in YPD at 25°C and transferred to a microscopy slide with a media-containing agarose patch. The cells were observed under PE spinning disc confocal microscope. The pictures were taken every 2 minutes.

Supplementary Table 1 The Strains used in this study

**Strain no**	**Genotype**
K699	*MATa,ade2-1,trp1-1,can1-100, leu2-3,112,his3-11,15,ura3,GAL,psi+*
K14061	*Mat a, Scc1PK9::KanMX*
K14710	*MAT a, Scc1PK9::KanMX, scc2-4*
K17407	*MATa, trp1::Smc3PK6::TRP1*
K17409	*MATa, trp1::smc3(E1155Q)PK6::TRP1*
K18719	*MATa/a/α/α, SCC1EGFP::HIS3, Mtw1-RFP:KanMX, ADE2*
K22390	*MATa, Scc1PK9::KanMX, scc2-45*
K22703	*MATa, trp1::smc3(K112Q, K113Q)PK6::TRP1*
K23308	*C.glabrata, Mat a, Scc1PK9::NatMX*

Supplementary Table 2 Summary of ChIP-seq

Figs	Experiments		Reads used in alignment	Uniquely mapped to S.cerevisiae	Uniquely mapped to C.glabrata	Mapped to both genomes
1	100%SacCer	IP	8649638	8149434	2742	333244
		WCE	5167495	4740954	472	249885
	50%SacCer	IP	8413598	5155020	2852480	270814
		WCE	6267558	2070269	3884251	187540
	100%CanGla	IP	7945771	11513	7681970	127372
		WCE	6302634	1327	6077692	162983
2	100%SacCer	IP	8187797	7448960	4375	391479
		WCE	9931526	8858474	258	578666
	80%SacCer	IP	6503237	4807985	1169076	282471
		WCE	14421988	9819225	3423828	701231
	60%SacCer	IP	7195107	3898658	2742289	299300
		WCE	7510027	3509427	3476955	344982
	40%SacCer	IP	5617248	1952501	3133843	194610
		WCE	8811110	2516456	5737817	351168
	20%SacCer	IP	11067924	1816853	8671375	293720
		WCE	4484097	554139	3699691	164315
	0%SacCer	IP	6300664	4358	5979285	134250
		WCE	4844809	357	4618598	166346
4	0’	IP	7543996	1715056	5263874	260471
		WCE	4799271	2149440	1824440	216780
	15’	IP	7681260	1881873	5247733	288333
		WCE	3835067	1227278	943047	129176
	30’	IP	7462394	3457095	3389708	413464
		WCE	4088149	1479460	1537002	150133
	45’	IP	8000705	5751973	1632536	390736
		WCE	4142400	2003686	880835	139048
	60’	IP	7901072	5364612	1880565	457384
		WCE	4037989	2015483	1067352	159223
	75’	IP	8742623	5425977	2627861	482907
		WCE	8109238	4156022	2370402	349516
	90’	IP	8281167	3709947	3925376	436316
		WCE	4037514	1927183	1221604	167245
	105	IP	8510608	4966486	2820996	513929
		WCE	3535912	1724098	1011956	141023
	120’	IP	7587642	5412167	1526342	448663
		WCE	3823932	2029288	950066	147404
6 and S7	Scc1	IP	7074281	212015	6488523	193026
		WCE	5190484	2658954	1952406	245065
	Scc1-PK9	IP	7807015	5325623	2034825	241734
		WCE	3974258	2208634	1288338	199282
	scc2-45	IP	11825641	1933071	9485933	80169
		WCE	11395420	4729218	6128951	228601
	WT	IP	6244312	3869289	2174583	71334
		WCE	11831591	5343407	6008495	191748
	scc2-4	IP	8477187	567386	7253166	44165
		WCE	11123673	2880422	7804500	237480

Figs	Experiments		Reads used in alignment	Uniquely mapped to S.cerevisiae	Uniquely mapped to C.glabrata	Mapped to both genomes
7	smc3 K112Q K113Q	IP	7382100	1496755	5311205	362779
		WCE	6513219	3593771	2397198	380127
	smc3 E1155Q	IP	7308322	2003371	4762496	374889
		WCE	9545913	5485553	3295436	569322
	Smc3	IP	5139660	2318971	2398303	325977
		WCE	7678693	4355238	2695373	466759
S5	0’	IP	8176945	1147587	6685318	154108
		WCE	7191787	2454652	4338134	270550
	15’	IP	7773362	2896497	4542125	147636
		WCE	5611224	2540812	2759138	191378
	30’	IP	9430721	7377225	1637029	224316
		WCE	5996454	2907268	2727325	207624
	45’	IP	9239164	7433012	1356368	224316
		WCE	6719251	3253102	3076632	235479
	60’	IP	8879382	6864050	1546378	329778
		WCE	7292148	3410023	3455545	271200
